# Reaction pathways, proton transfer, and proton pumping in ba3 class cytochrome c oxidase: perspectives from DFT quantum chemistry and molecular dynamics

**DOI:** 10.3389/fchem.2023.1186022

**Published:** 2023-12-22

**Authors:** Louis Noodleman, Andreas W. Götz, Wen-Ge Han Du, Laura Hunsicker-Wang

**Affiliations:** ^1^ Department of Integrative Structural and Computational Biology, The Scripps Research Institute, La Jolla, CA, United States; ^2^ San Diego Supercomputer Center, University of California San Diego, La Jolla, CA, United States; ^3^ Department of Chemistry, Trinity University, San Antonio, TX, United States

**Keywords:** cytochrome c oxidase, electron transport chain, bioenergetics, reaction pathway, proton transfer, proton pumping, DFT calculations, MD simulations

## Abstract

After drawing comparisons between the reaction pathways of cytochrome c oxidase (CcO, Complex 4) and the preceding complex cytochrome bc_1_ (Complex 3), both being proton pumping complexes along the electron transport chain, we provide an analysis of the reaction pathways in bacterial ba_3_ class CcO, comparing spectroscopic results and kinetics observations with results from DFT calculations. For an important arc of the catalytic cycle in CcO, we can trace the energy pathways for the chemical protons and show how these pathways drive proton pumping of the vectorial protons. We then explore the proton loading network above the Fe heme a_3_–Cu_B_ catalytic center, showing how protons are loaded in and then released by combining DFT-based reaction energies with molecular dynamics simulations over states of that cycle. We also propose some additional reaction pathways for the chemical and vector protons based on our recent work with spectroscopic support.

## 1 Introduction

**FIGURE 1:**
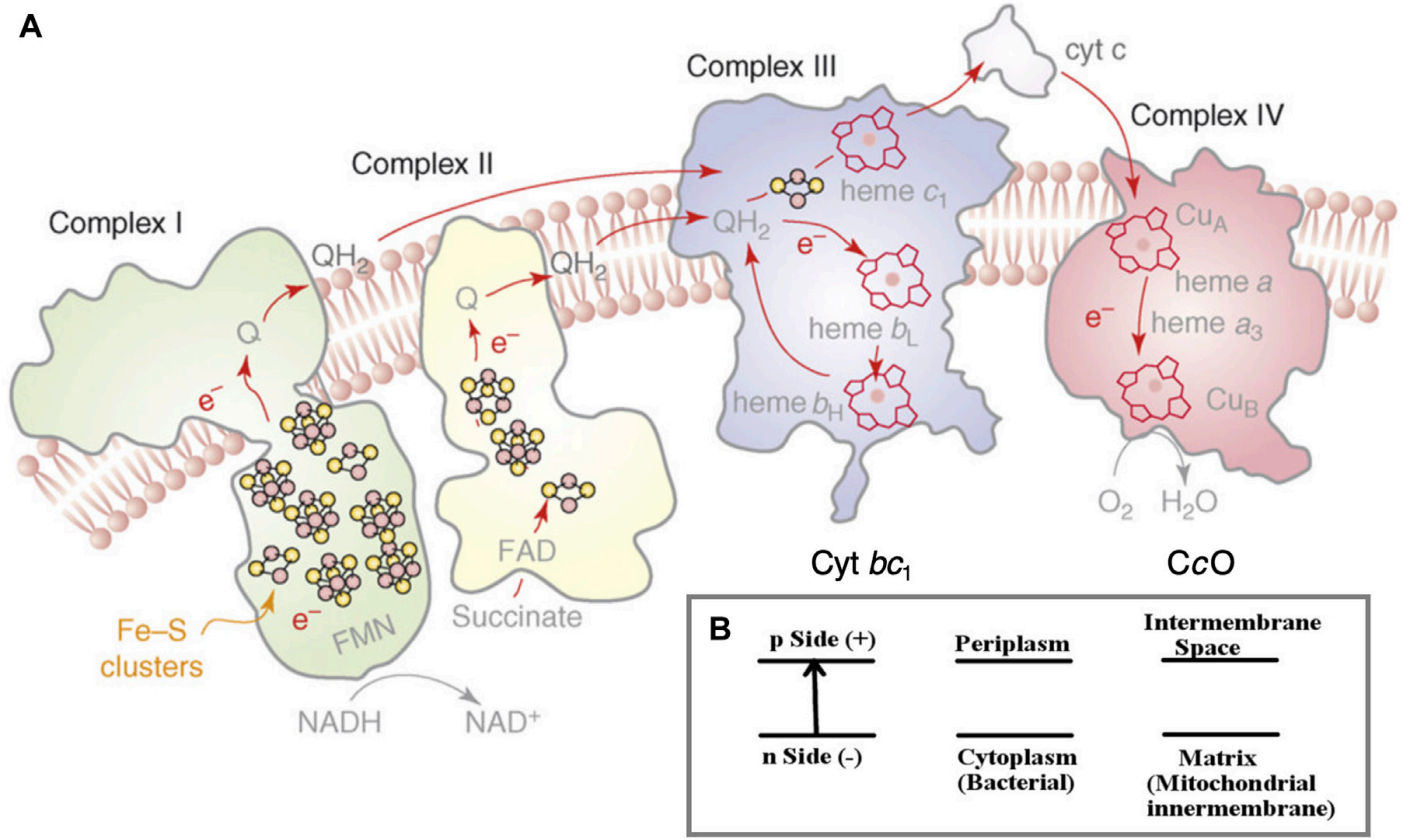
**(A)** Schematic of the four integral membrane protein complexes I–IV in the electron transport chain in mitochondria and aerobic bacteria. The inner (lower) region below the membrane as depicted is the n-side (−) corresponding to the matrix space in the mitochondria and to the cytoplasm in bacteria. The outer (upper) region above the membrane depicted is the p-side (+), intermembrane space in mitochondria, and periplasm in bacteria. The electrons are delivered to cytochrome c oxidase (CcO) by cytochrome c (cyt c), as shown on the p-side of the membrane. Reprint with permission from Figure 1 of Rouault and Tong (2008), copyright 2008 Elsevier. **(B)** The electrochemical potential gradient across the membrane.

The flow of electrons through an ordered series of proteins embedded in the inner membranes of mitochondria and in aerobic bacteria provides the driving force for proton transfer across these membranes (Nicholls and Ferguson, 2002; Rouault and Tong, 2008; Noodleman et al., 2020). (Figure 1A) In an intact inner membrane containing all the relevant integral membrane protein complexes (Complexes 1–4), with sufficient reducing equivalents from NADH (a two-electron–one-proton donor plus an added proton (2e^−^, 2H^+^)), and with access to adequate molecular oxygen O_2_ (the terminal electron–proton acceptor for the chemistry) in complex 4, a substantial transmembrane electrochemical potential difference is generated. The negative potential side is inside and the positive potential side outside of the membrane, the proton flow being from inside to outside (Noodleman et al., 2020) (Figure 1B). This potential difference in turn drives proton transfer back through the neighboring protein complex 5 (across the membrane in the opposite direction), turning a rotor within complex 5, and produces ATP from ADP plus inorganic phosphate (Pi) by a dehydration reaction.

## 2 Comparing cytochrome bc1 and cytochrome c oxidase (complex 3 with complex 4)

To have a frame of reference for the mechanism and significance of reaction steps in complex 4 (cytochrome c oxidase), we compare some broad features of complex 4 with those in complex 3 (cytochrome bc_1_). In the electron transport chain (ETC), protons are pumped across the membrane at complexes 1, 3, and 4 (Figure 1A). Already at complex 1, two electrons and two protons (2e^−^, 2H^+^) are added to the mobile carrier molecule, oxidized ubiquinone (Q), to produce the 2e^−^ reduced form (QH_2_), a two-electron carrier. Complex 2, which does not pump protons, still adds reducing equivalents to Q, producing additional QH_2_. In its reduced form, QH_2_ readily flows through the membrane and is mobile as well within protein complex 3, being able to transit from the upper to the lower part of the protein in both its oxidized form Q and reduced form QH_2_. Both forms Q and QH_2_ are uncharged. Furthermore, the long hydrophobic sidechain of the quinone makes it amphiphilic overall, so it can move easily between hydrophilic and hydrophobic regions.

Complex 3 acts as a transducer of electron flow, converting the 2e^−^ transfer by QH_2_ into 1e^−^ transfers within complex 3, on to cytochrome c, the mobile protein carrier for 1e^−^ delivery to complex 4. A relevant question for the mechanism of complex 3, and more generally across biological systems, is how this and related electron transduction systems work. The key feature is a bifurcation (splitting) of electron transfer between a high potential pathway (here through the Rieske protein subunit and on to cytochrome c_1_ within subunit b) and a low potential pathway (through cytochrome b_L_ and ending with cytochrome b_H_, from lower to higher redox potential). The initial reaction works in two steps: 1): QH_2_ → Q^−^ (radical) + 2H^+^ + 1e^−^; then, an oxidation step occurs, 2): Q^−^ (radical) → Q + 1e^−^, with the acceptors having corresponding redox potentials, high potential for step 1 and low potential for step 2. The high potential pathway of step 1 has as its first acceptor the nearby [2Fe-2S] cluster of the Rieske protein subunit. The one electron (1e^−^) transfer via this pathway is required to produce the high-energy Q^−^ (radical) state.

**FIGURE 2:**
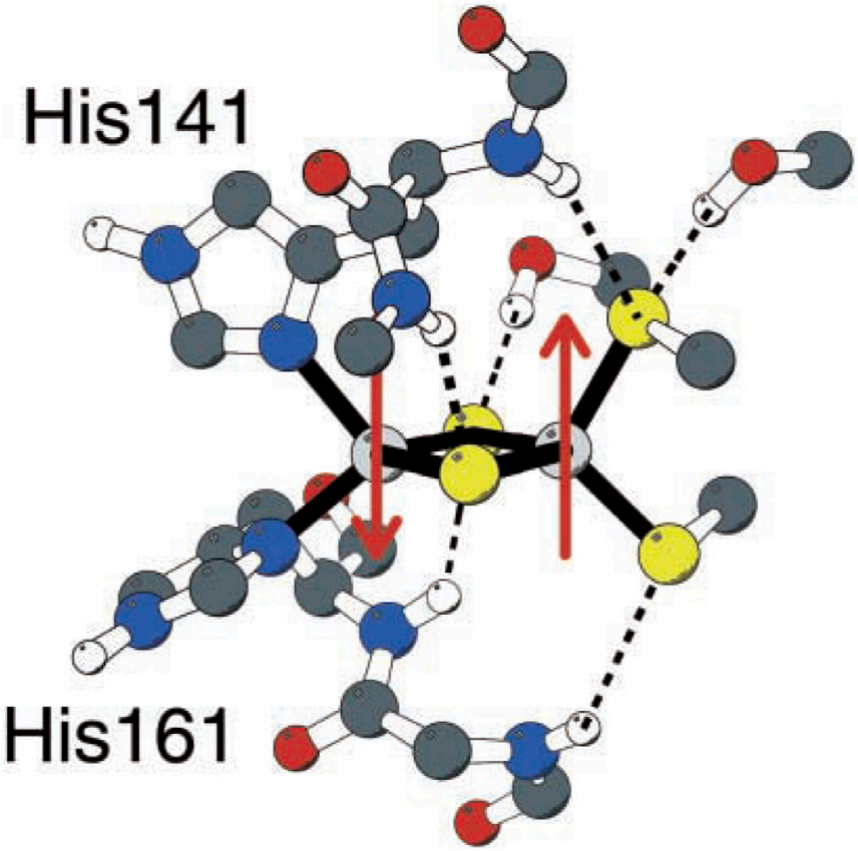
Rieske [2Fe-2S] center of the Rieske protein subunit in complex 3. Non-polar hydrogen atoms are omitted for clarity. Reproduced from Figure 1 of Ullmann et al. (2002), copyright 2002 Springer.

Unlike typical [2Fe-2S] clusters with four cysteines 4(SCys^−^) linked to 2Fe, the Rieske FeS center has 2(SCys^−^) bound to one Fe and 2(NHis) bound to the alternate Fe (Figure 2) (Ullmann et al., 2002). So how does the relatively high redox potential of the Rieske [2Fe-2S] cluster and protein arise? It was discovered that the Rieske protein has a pH-dependent redox potential in complex 3 systems attributed to additional proton binding on 1e^−^ reduction to the *ɛ*-nitrogen of the histidines, those N atoms not directly bound to Fe. The pH dependence of the redox potential was examined in detail experimentally in the Rieske protein fragment isolated from *Thermus thermophilus* (Tt) bacteria (Zu et al., 2001), and this property is similar in the mitochondrial (bovine) Rieske protein fragment (Kuila et al., 1992; Link et al., 1992; Baymann et al., 1999). Independently at about the same time, our group demonstrated that one electron reduction of the Rieske cluster involves a coupled single-proton transfer using a combination of density functional theory (DFT) calculations on the Rieske [2Fe-2S] cluster and electrostatic methods from the Poisson–Boltzmann theory for the surrounding protein (Ullmann et al., 2002). Analysis of the pH dependence of the redox potential of the Rieske protein fragment (Tt) gives experimental effective p*K*_a_ values for two sites in the oxidized form p*K*_a_ = 7.8 and 9.6 and for the reduced form p*K*_a_ = 12.5 (approximately) for both sites from the fit. If we consider protonation of an empty site at a typical pH = 7 on one of the two His during 1e^−^ reduction, the calculated Δ*G* = 1.37 × (7–12.5) kcal/mol = −7.54 kcal/mol, equivalent to a positive shift in the redox potential equal to +330 mV. This clearly is a large contributor to the high redox potential of the Rieske protein connecting to the high potential pathway. Both pH-dependent NMR and chemical modification species support the deprotonation of histidine 154 (Tt) (Hsueh et al., 2010; Konkle et al., 2010), the first site deprotonated with increasing pH in the oxidized Rieske protein.

However, we encounter another more subtle issue. In the oxidized form of Tt, the lowest affinity site still has a p*K*_a_ = 7.8. At pH = 7, the fractional protonation (occupation) of that site is 0.86 from the Henderson–Hasselbalch equation, so protonation of that site from the reduced quinone QH_2_ will be strongly inhibited, unless the site is previously emptied by another mechanism. Rieske proteins isolated from other cytochrome bc_1_ species show similar oxidized p*K*_a_ values, including from bovine mitochondria (Link et al., 1992) and *Rhodobacter sphaeroides* (Rs) cyanobacteria (Zu et al., 2003). We noticed that all the pH-dependent redox measurements were carried out on the isolated Rieske fragment and after a search found that in the intact bc_1_ enzyme, there is a critical lysine residue with a positively charged mobile sidechain in the cytochrome b subunit, very near the Rieske iron–sulfur cluster. Assuming this lysine sidechain coordinates close to the histidines, as is feasible in the oxidized redox state, this interaction will favor deprotonation of the lower-affinity histidine site. This lysine is expected to move away from the histidines in the reduced state, where the p*K*_a_ values are much higher. In a collaboration with two experimental groups, this proposal was successfully tested (Francia et al., 2019). This lysine sidechain was found to be isostructurally conserved across many species, including both prokaryotes and eukaryotes. Furthermore, experimental tests on *Rhodobacter capsulatus* (Rc) cyanobacteria, acting in photosynthetic mode, showed that both the native lysine residue and an arginine mutant in that position are fully active for both electron transfer and proton pumping, while anionic amino acid residues in that position are completely inactive, and neutral amino acids have diminished activity. Rc cyanobacteria are functionally and structurally similar to Rs bacteria.

Once the Rieske cluster is 1e^−^ reduced and protonated by 1H^+^, the following electron and proton transfer involves a large-scale movement of the [2Fe-2S] cluster and protein (Rieske head group) by several angstroms to dock to a site near the embedded cytochrome c_1_ cofactor. The proton proceeds through the exit path to the intermembrane region, while the single electron is transferred from cytochrome c_1_ to cytochrome c, which is the 1e^−^ donor to cytochrome c oxidase, see Figure 1A and the discussion and Figure 2 in Noodleman et al. (2020). Thus, the proton transfer that occurs in complex 3 in the Rieske protein results from a deprotonated histidine accepting an H^+^ along with the electron as the QH_2_ is oxidized to semiquinone. The second proton is transferred to a different histidine (not within the Rieske cluster).

The structure and mechanism of complex 4 are quite different. There are no mobile electron and proton carrier molecules like ubiquinone or the Rieske protein head group. Instead, as we will see, water plays a major role, with smaller changes in protein geometry. There are no bifurcations of electron flow in CcO. Instead, there are bifurcations of proton flow, which are connected with oxygen binding and electron transfers. There are also differences in the mechanisms and function of the bacterial ba_3_ class of CcO, exemplified by *Thermus thermophilus*, and aa_3_ class CcO found in other aerobic bacteria and in mitochondria.

## 3 Structure, mechanism, and function of cytochrome c oxidases

**FIGURE 3:**
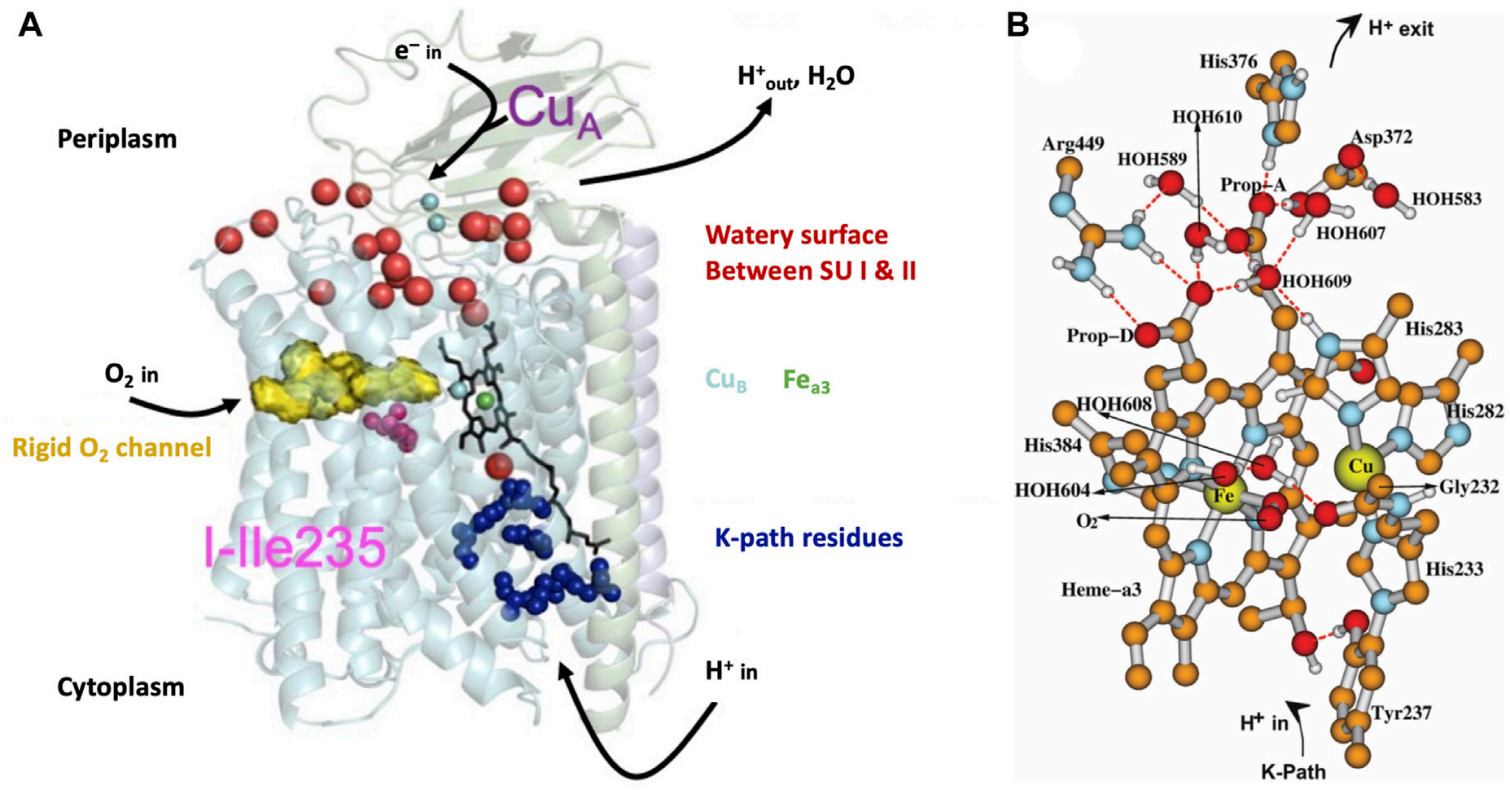
**(A)** Schematic of cytochrome ba_3_ with noted functionalities. SU is a subunit. Reproduced from Figure 1 of Noodleman et al. (2014), copyright 2014 American Chemical Society (further permissions for reusing this figure should be directed to the ACS). **(B)** Dinuclear center (DNC) of cytochrome c oxidase consisting of heme Fe_a3_, Cu_B_, and relevant residues and water molecules in the vicinity. Shown is a structural model for the adduct state A of the DNC in which molecular oxygen O_2_ coordinates to Fe_a3_. Some apolar hydrogen atoms are omitted for clarity. Reproduced from Figure 3 of ref. Han Du et al. (2019), copyright 2019 American Chemical Society (further permissions for reusing this figure should be directed to the ACS).

In Figure 3A, we present a structural global representation of the ba_3_-type cytochrome c oxidase from *Thermus thermophilus* (Tt) (Chang et al., 2009). Electrons enter by transfer from the one-electron (1e^−^) protein carrier molecule cytochrome c and are passed to Cu_A_, which is a dinuclear Cu cluster. From Cu_A_, electrons transfer via tunneling through the protein to the low-spin mononuclear heme Fe_b_ (in this Figure hidden behind the DNC Fe_a3_ heme) and then on to the dinuclear center (DNC) [also called the binuclear center (BNC)]. The DNC consists of heme Fe_a3_ with an axial histidine ligand, a nearby Cu site called Cu_B_ bonded to three histidines, and the surrounding protein and water molecules (see Figure 3B). One of these histidines below Cu(B) has a special covalent linkage to a tyrosine (N-C) via their sidechains (His233 to Tyr237 in Tt ba_3_). This linkage is universal in both ba_3_- and aa_3_-type CcOs and has not been found so far in proteins other than CcO. Protons enter the CcO protein from the cytoplasm connected to the membrane inner surface, as schematically shown in Figure 3. CcO proteins are embedded in the inner membrane both in the mitochondria and in bacteria. The transfer of protons from the inside (n-side, negative) to the outside (p-side, positive) of the membrane and the opposite transfer of electrons from outside to inside produce an electrochemical potential difference across the membrane (Figure 1B). Importantly, this potential difference arises not only from proton pumping but also from the catalytic reaction chemistry cycle, which removes positive charge (protons) from the inside and negative charge (electrons) from the outside and combines these with O_2_ to produce water. The overall reaction is as follows:

$${\text{O}}_{2}+4{\text{e}}^{-}+\left(4+n\right){\text{H}}^{+}\left(\text{in}\right)\to 2{\text{H}}_{2}\text{O}+n{\text{H}}^{+}\left(\text{out}\right).$$ (1)

Here, 4e^−^ and 4H^+^ are used in the chemical reaction cycle, and *n*H^+^ is pumped from the inside to outside. O_2_ enters via a hydrophobic channel from the membrane’s interior, passing near Cu_B_ and binding to Fe_a3_ starting from its reduced Fe^2+^ state. (see Figure 3A) In the equation above, the total amount of charge transferred is *Q* = 4 + *n* electron charge equivalents per catalytic cycle, where *n* is between 2 and 4. To have a sufficient number of electrons to produce a full reaction cycle from the oxidized enzyme, 4e^−^ must be added to CcO in 1e^−^ steps originating sequentially from four Cyt c molecules. At that fully reduced (4e^−^) stage, Cu_A_ has redox state Cu^+^–Cu^+^ for the copper cluster starting from the mixed-valence oxidized state Cu^1.5+^–Cu^1.5+^, Fe_b_ is in the Fe^2+^ state, 1e^−^ reduced from the oxidized Fe^3+^ state, and the DNC is also reduced, overall by 2e^−^, meaning ${\text{Fe}}_{\text{a}3}^{2+}$ and ${\text{Cu}}_{\text{B}}^{+}$ (reduced state R). A comprehensive discussion of the reaction cycle and the intermediate states can be found in Noodleman et al. (2014).

**FIGURE 4:**
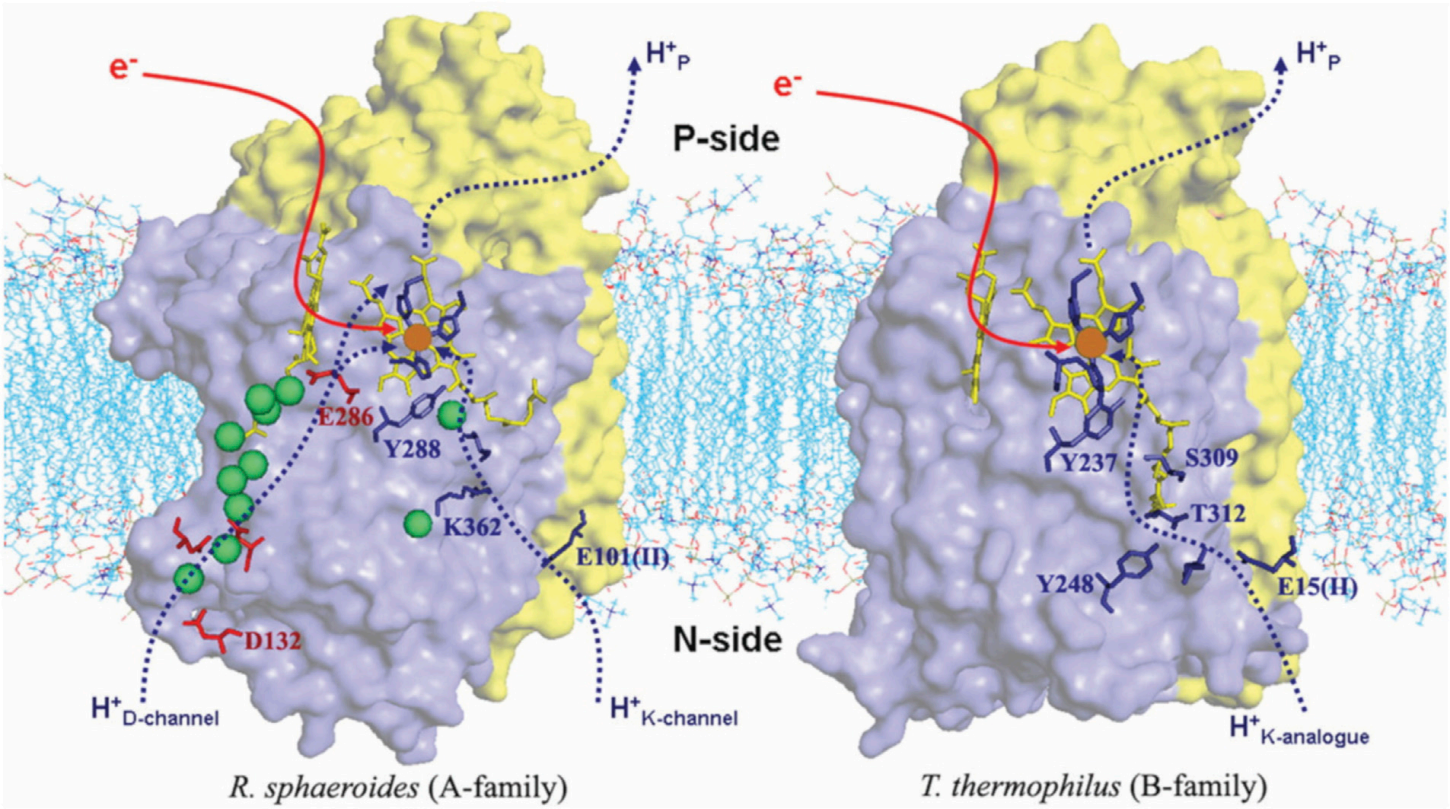
Comparison of the proton delivery pathways for the A- and B-types of CcO. Green spheres are water molecules. Reproduced with permission from Figure 3 of Chang et al. (2009).

Figure 4 shows the comparison of structures and proton entry paths for A- and B-family CcOs, with *R. sphaeroides* (A-family, aa_3_-type) and *T. thermophilus* (B-family, ba_3_-type) as examples (Chang et al., 2009; Noodleman et al., 2020). There are two different entry paths (D and K paths) to the DNC in aa_3_ enzymes and only one (K path analog) in ba_3_ enzymes (Wikström et al., 2018). For the D-path in *R. sphaeroides*, the proton entry involves an Asp132 (D132), which takes a proton from the cytoplasmic region (Figure 1B) (analogous to the matrix space of the mitochondrial inner membrane in animals). This proton then passes through a series of sites, including several water molecules (green spheres as depicted) and protein sidechains, until it reaches the Glu286 (E286). This residue sidechain is at or near a branch point, where protons may go either into the reactive region where O_2_ binds and is transformed or protons may be diverted for proton pumping, depending on the state of the reaction cycle and other conditions. For the other proton input path, in the aa_3_ family, a Glu (E101 of subunit II in *R. sphaeroides*) picks up the proton from the cytoplasm (n-side), and then this proton is transported from site to site, eventually reaching the special tyrosine (Y288). A lysine residue (K362) plays an important role in the transport through the K path in aa_3_ enzymes. In comparison, in the B-family (ba_3_ as in *T. thermophilus*), there is no D-path for protons into the reactive complex region or for proton pumping, but only a single-proton input path starting at the Glu (E15 of subunit II in *T. thermophilus*) and then leading into the reactive region via the special tyrosine (Y237).

Table 1 summarizes the number of vectorial and chemical protons that are transported via the different proton channels in aa_3_- and ba_3_-type enzymes. From proton pumping experiments on cytochrome ba_3_ in lipid vesicles, Rauhamaki and Wikstrom found that two or more vector protons (three or four) are pumped per catalytic cycle, compared to four in cytochrome aa_3_, but with considerable uncertainties (Rauhamäki and Wikström, 2014). They attribute this variability either to the greater sensitivity of ba_3_ enzymes to higher membrane potentials or to increased proton backflow compared to aa_3_ enzymes plus measurement difficulties. Table 2 shows the different modes of transport feasible for electrons and for protons as well as their respective ranges for single tunneling events.

**TABLE 1:** Proton and electron transport pathways and mechanisms in CcO. Reproduced from Noodleman et al. (2020).

Pathway	Chemical	Vector	Total
aa_3_-type (bacterial and mitochondrial)			
K-path	2	0	2
D-path	2	4	6
All paths	4	4	8
ba_3_-type (bacterial)			
K-path	4	2–4	6–8
D-path	0	0	0
All paths	4	2–4	6–8

Differences in proton channels and number and types of transported protons in bacterial and mitochondrial CcO.

**TABLE 2:** Proton and electron transport pathways and mechanisms in CcO. Reproduced Noodleman et al. (2020).

Type	Mechanism
Electron	Tunneling—max range approximately 11–14 Å
Electron	Transport on mobile carrier
Proton	Tunneling—max range approximately 1 Å
Proton	Transport on mobile carrier
Proton	Transport on amino acid sidechain
Proton	Transport by diffusion on water (H_2_O as carrier)
Proton	Grotthuss process—-fast proton transfer along chains of water molecules, also in combination with amino acid sidechains (bucket brigade mechanism)

Modes of proton and electron mobility in proteins and membranes.

### 3.1 Catalytic reaction cycle in the DNC

**FIGURE 5:**
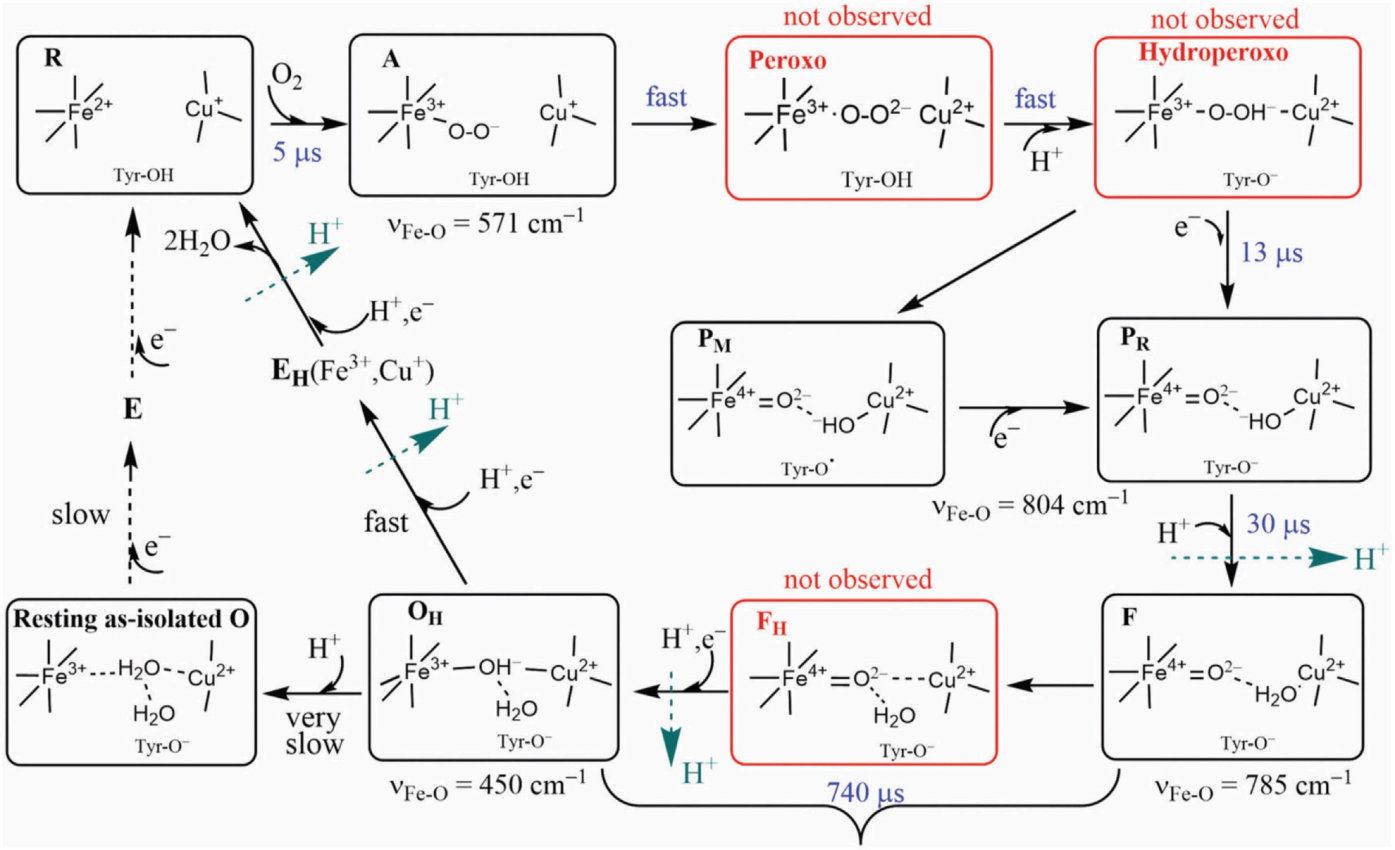
Intermediate states of the DNC in the catalytic cycle of CcO. States A, P_M_/P_R_, F, and O_H_ were identified by resonance Raman experiments on A-family oxidases. Although the peroxo, hydroperoxo, and F_H_ states (red frames) were not observed experimentally, they may exist for a short time. Tyr is the special Y288 in aa_3_ and Y237 in ba_3_ enzymes. Reproduced with permission from Figure 6 of Noodleman et al. (2020).

We will focus on the major arc of the DNC catalytic reaction cycle from the reduced state R binding to O_2_ (state A) through several intermediates to the reactive oxidized state, labeled O_H_, prepared for the next redox, proton pumping, and oxygen binding cycle. (In the “fully reduced enzyme,” all metal sites are reduced, Fe^2+^ or Cu^+^.) From the viewpoint of the DNC, additional electrons, where needed, are supplied directly by Fe_b_ (in the ba_3_ enzyme), and the Cu_A_ dimer then reduces Fe_b_ for the next 1e^−^ donation to ${\text{Fe}}_{\text{a}3}^{3+}$ (or to ${\text{Fe}}_{\text{a}3}^{4+}$ ) or ${\text{Cu}}_{\text{B}}^{2+}$ . The Cu_A_ cluster and Fe_b_ are resupplied by electrons from Cyt c as needed, but always in single-electron transfer steps. The DNC catalytic reaction cycle is depicted in Figure 5 (Noodleman et al., 2020). Over a full cycle from State R and returning to State R, four electrons (4e^−^) are taken up, as shown. Four chemical protons are also taken up from input channels. In Figure 5, the sequence of states and transitions shows a close similarity between the ba_3_ class and aa_3_ class enzymes from optical difference spectroscopy, but with some differences in O_2_ binding affinity and kinetics through the cycle. First, we have a few comments about the sequence of intermediates, kinetics, and other physical parameters portrayed in Figure 5. The kinetic rate constants for various transitions depicted in Figure 5 are taken from the work of Einarsdottir et al. (Szundi et al., 2010) using optical difference spectroscopy on ba_3_ CcO intermediates from Tt in the detergent to track the electron transfer from one metal cofactor (reduced *versus* oxidized states) to the next. (Their group and others have done comparable work on aa_3_ class enzymes, where there is some loss in accuracy for kinetics parameters due to the overlapping optical difference absorption bands for heme a and heme a_3_). For our purpose, the most important aspects of the reaction cycle kinetics are the very fast rates from R → F and the much slower rates from F → O_H_ (Bloch et al., 2004). State O_H_ is an activated state which promotes the following electron transfer to generate State E_H_ and, therefore, activates the next catalytic cycle (Bloch et al., 2004). If the electron transfer occurs too slowly, O_H_ decays to the “resting” State O, which is more difficult to reduce. While these differences in kinetics are now evident, the structural differences between States O_H_ and O are not, either in aa_3_-type or in ba_3_-type CcOs. We used detailed DFT modeling (te Velde et al., 2001; van Lenthe and Baerends, 2003; Baerends et al., 2017) of different potential geometric and electronic structures, including relative energies and predicted *versus* observed Mössbauer parameters, to propose likely structures for State O compared to State O_H_ (Han Du et al., 2020; Han Du et al., 2022). Important work involving spectroscopy and kinetics continues on this problem.

**FIGURE 6:**
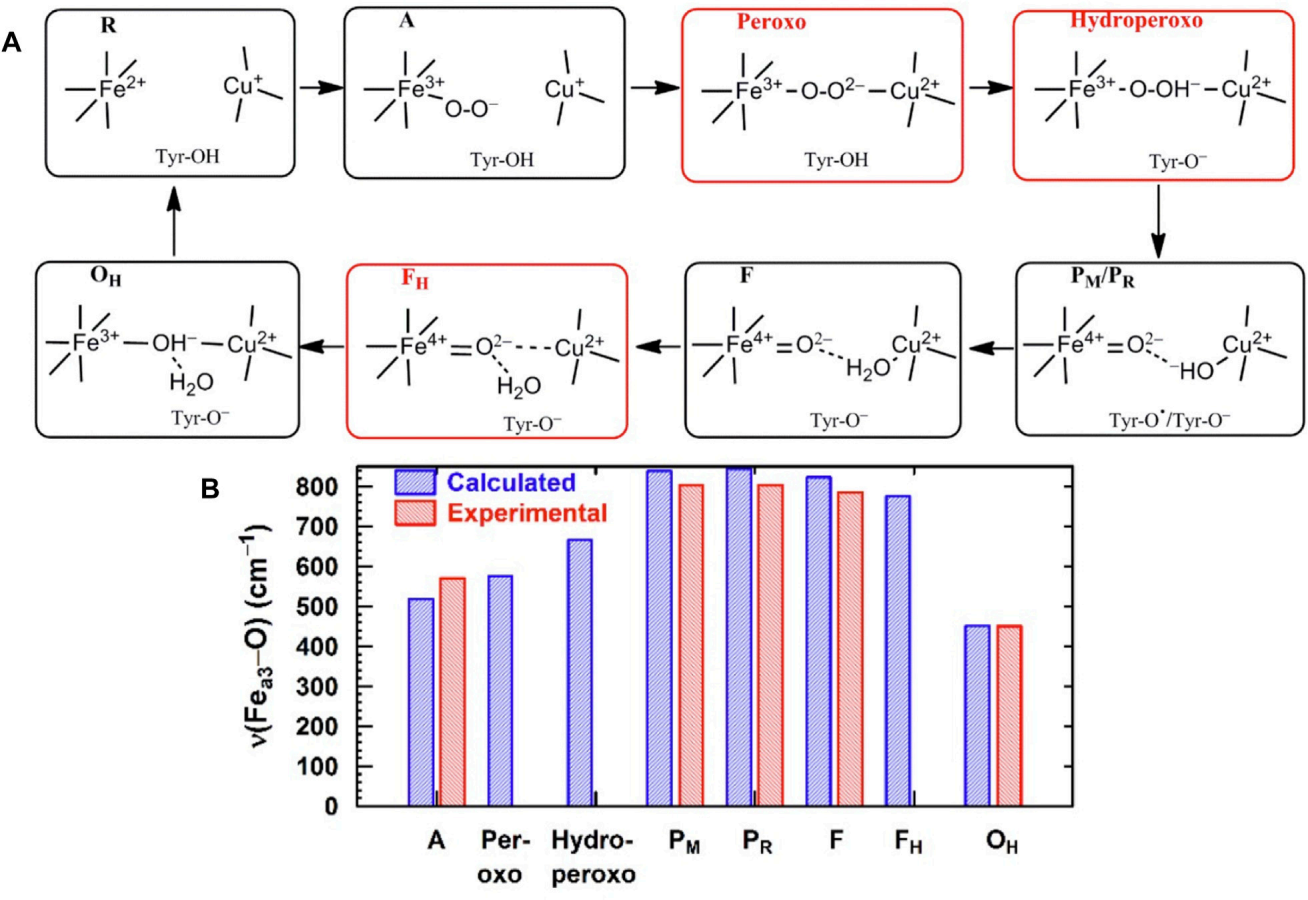
**(A)** Simplified map of the intermediate states of the DNC in the catalytic cycle of CcO shown in Figure 5. Reproduced from Figure 1 in Han Du et al. (2019), copyright 2019 American Chemical Society (further permissions for reusing this figure should be directed to the ACS). **(B)** Comparison of Fe_a3_–O/O–O vibrational frequencies for states along the reaction cycle of CcO obtained from DFT calculations on DNC cluster models and from resonance Raman experiments.

**FIGURE 7:**
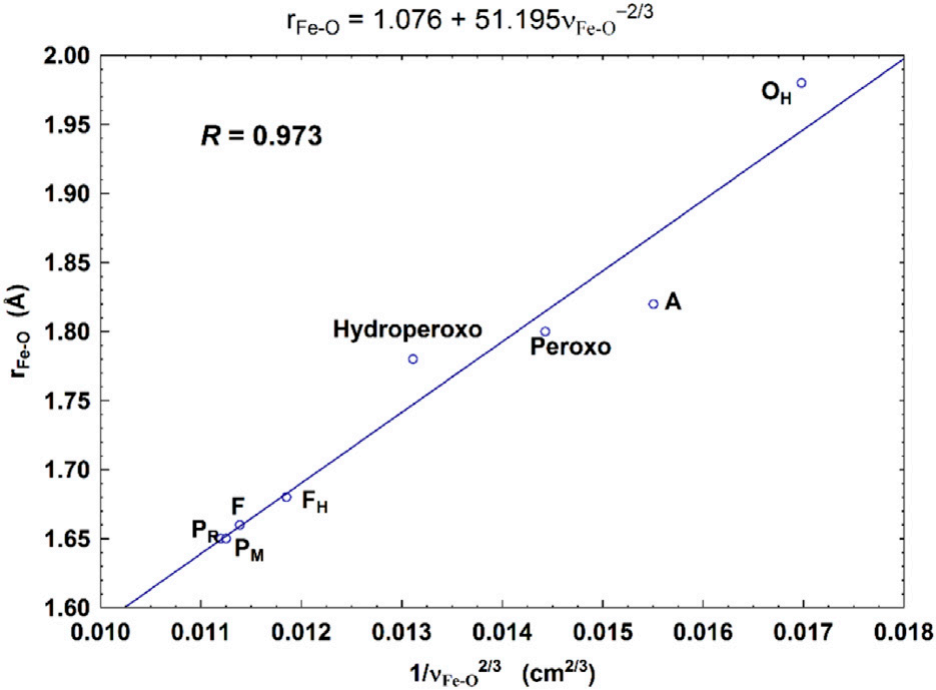
Correlation between the calculated Fe_a3_–O bond lengths (*r*_Fe-O_, Å) and the corresponding Fe_a3_–O stretch frequencies (1/ $\nu_{\text{Fe}-\text{O}}^{2/3}$ , based on Badger’s rule) for eight DNC state structures. Reproduced from Figure 7 in Han Du et al. (2019), copyright 2019 American Chemical Society (further permissions for reusing this figure should be directed to the ACS).

For CcO in detergents [in Einarsdottir’s and related work (Siletsky et al., 2007; Szundi et al., 2010)], there is no net proton pumping, no electrostatic potential is developed because there is no membrane, and there is no net sidedness (orientation) in physical space for the CcO protein molecules. In the presence of a membrane, as, for example, in experiments where CcO proteins are embedded in lipid vesicles with preferred directionality, the transfer rates from state to state are slower because a membrane potential is developed producing added energy barriers for proton pumping and barriers involving the chemical reaction cycle steps as well. We also follow the chemical reaction cycle with resonance Raman vibrational spectroscopy from the work of Rousseau et al. (Egawa et al., 2012; Ishigami et al., 2015). The observed Fe–O stretch frequencies for the series of States are shown in Figure 5 for an aa_3_ class enzyme; ba_3_ intermediates and Fe–O frequencies are expected to be similar. We have calculated DFT Fe–O frequencies for our ba_3_ quantum cluster models (Han Du et al., 2019) showing good correspondence with the measured frequencies in aa_3_ (Figure 6B), and the sequence of intermediates from DFT tracks well with the observed sequence of different frequencies from resonance Raman spectroscopy. Figure 7 shows an excellent linear correlation between the DFT-calculated Fe–O distances over the reaction pathway with a function of the DFT-calculated Fe–O frequencies (Han Du et al., 2019) [an empirical inverse power law correlation called Badger’s rule (Badger, 1935)]. A simplified map of the different states through the cycle is also provided (Figure 6A) (Han Du et al., 2019). In the bar graph comparing the DFT-calculated *versus* experimental resonance Raman frequencies, we want to emphasize some points. There are no experimentally observed resonance Raman frequencies for peroxo or hydroperoxo states, probably due to the very fast kinetic steps going through these intermediates starting from State A to states P_M_ and P_R_. In related synthetic systems with peroxo or hydroperoxo bridges, the measured resonance Raman frequencies do fit those calculated here by DFT for CcO well. The experimental absence of these two states does raise the question of which is, in fact, present and in what form?

State A, where O_2_ binds to ferrous Fe (see the prior State R) to form the “ferrous-adduct,” has an important electronic structure and illustrates some of the difficulties in obtaining accurate electronic structure descriptions. Figure 3B shows the geometric structure of State A. State A also sets the stage for the subsequent rapid sequence of reactions. During the formation of State A from the reduced State R, where the high-spin ferrous (heme a_3_) metal ion Fe^2+^ reacts with the high-spin triplet ground state of O_2_, effectively, there is a 1e^−^ transfer from Fe^2+^ to O_2_, with partial covalent-ionic bonding between low-spin ferric Fe^3+^ and low-spin superoxide radical anion ${\text{O}}_{2}^{-}$ . Substantial negative charge transfer occurs from the Fe^2+^ to the O_2_, but less than one full electron. The calculated Mayer bond order (Mayer, 1983) is 0.48 for the Fe–O stretch in state A, which slowly increases to 0.54 and 0.59 for the following peroxo and hydroperoxo states, while the O–O Mayer bond order successively decreases from 1.28 for State A to 1.06 for peroxo and 0.911 for hydroperoxo. As expected, the Fe–O bond strengthens and shortens through this sequence of states, while the O–O bond weakens and lengthens in preparation for breaking. The calculated Fe–O and O–O stretch frequencies follow this same pattern, see Figure 7. The DFT-predicted Fe–O stretch frequency for state A of 518 cm^−1^ deviates by approximately 10% from the experimental value of 571 cm^−1^, while the DFT-calculated O–O stretch for superoxide is accurate to approximately 5% compared with experiments in comparable systems, like oxymyoglobin. While the calculated Fe–O stretch frequency error is the largest percentage for the entire observed reaction cycle, this error is still fairly small. The O_2_ binding process is intrinsically complex, involving 1e^−^ oxidation of the Fe^2+^ site, reduction of O_2_, and weakening of the *π* bond in forming the superoxide radical anion ${\text{O}}_{2}^{-}$ , conversion of both fragments into low-spin S = 1/2 forms, and covalent bonding with partial back charge transfer of electron density from ${\text{O}}_{2}^{-}$ to Fe^3+^ in the porphyrin site. We have shown (Noodleman et al., 2020) that the overall reaction here involves some compensating errors, particularly involving the oxidation of the Fe^2+^ complex and the partial bond breaking of the O_2_ *π* bond. Proceeding to the next steps of the catalytic cycle, after the assumed 1e^−^ transfer to ${\text{O}}_{2}^{-}$ from Cu^+^ → Cu^2+^ in Cu_B_ to form the Fe^3+^–(O–O)^2−^–Cu^2+^, peroxo bridge state, the following proton transfer from the special tyrosine (Tyr-OH) shows a significant positive energy difference (uphill) to form the hydroperoxo bridge state, calculated by DFT, approximately 12 kcal/mol higher than that of the peroxo state. By contrast, when we followed the actual O–OH bond cleavage of the hydroperoxo state along the O–O reaction coordinate, this energy barrier is quite small, approximately 1–2 kcal/mol (Han Du et al., 2016). The DFT-calculated (approximate) transition state has an O–O bond length near 1.7 Å, which then generates the final state P_M_, with the final fourth electron to break the O–O bond derived by 1e^−^ transfer from the special tyrosine (Tyr237^−^ anion in *T. thermophilus*) through the covalently linked His233 to Cu^2+^ and on to O_2_.

Let us now examine in detail some of the structural features of the DNC from *T. thermophilus* using our DFT calculated structural model for the Fe-superoxide-bound State A (see Figure 3B). Near the top of the DNC is the sidechain ring of His376, a small water pool, the sidechain of Asp372, and the heme a_3_ proprionate sidechain (Prop-A). These residues form the start of the proton-loading network (PLN) for the vector proton transit. His376 is central to this network and forms the proton-loading site for vector protons, as our group and others have proposed. In the hydroperoxo state, the Asp372 is thought to be protonated, as discussed later, and His376 is doubly protonated, forming a histidine cation with a high p*K*_a_, approximately p*K*_a_ = 11.0 from our DFT calculations (OLYP potential with D3 dispersion) (Han Du et al., 2016). After O–O bond cleavage in the hydroperoxo state to form State P_M_, the His376^+^ p*K*_a_ remains similar, and DFT-calculated p*K*_a_ = 11.8. The next steps are formation of State P_R_ by “external” 1e^−^ transfer from heme b Fe^2+^ and then proton transfer to Tyr237. The proton transfer source is a glutamate/glutamic acid at the starting point of the K pathway into the DNC along a hydrogen-bonded chain of residues and water molecules to the Tyr237. At this stage, after net (1e^−^,1H^+^) transferred to the Tyr237 neutral radical, we have calculated the His376^+^ p*K*_a_ and again found it to be high, p*K*_a_ = 9.2 (Han Du et al., 2016). These three states, hydroperoxo, P_M_, and P_R_ after Tyr237 protonation (neutral) form convenient reference states to examine proton transfers in the proton-loading network and exit paths.

**FIGURE 8:**
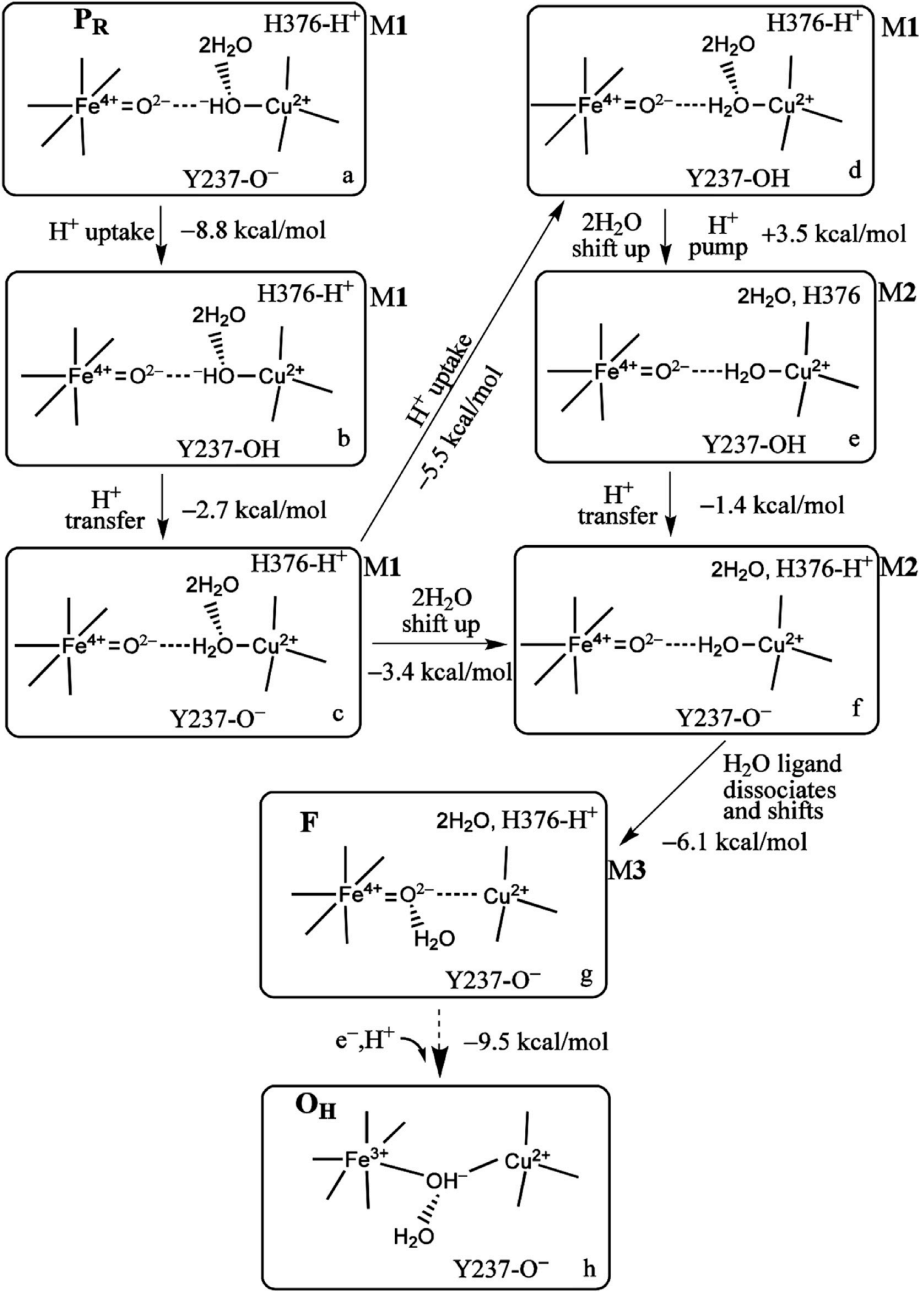
Proton uptake/pumping processes during the P_R_ →F →O_H_ transition. The two water molecules are HOH604 and HOH608 from Figure 3B. The Δ*G* (kcal/mol) change at pH = 7 (Δ*G* =1.37 (pK_*a*_ −7) between two different protonation states and the energy difference corrected by zero point energy between two tautomers are shown. The indices (a through h) label the various redox, protonation, and structural states as discussed in the text. Reproduced from Figure 4 in Han Du et al. -2018), copyright 2018 American Chemical Society (further permissions for reusing this figure should be directed to the ACS).

First, however, we explore the state-to-state analysis from P_R_ → F → O_H_ in more detail, which will illuminate the driving force for proton pumping at this stage of the cycle, see Figure 8 (Han Du et al., 2016). In Figure 8, the number and types of intermediates amplify on those summarized in Figure 5. The number of intermediates from P_R_ → F → O_H_ has been expanded with more detailed typing. These states are labeled from a → b → c → d → e → f → g → h. We also distinguish three types of hydration models near the DNC central reaction chamber, M1, M2, and M3. These models contain explicit quantum-described water molecules. The first hydration model (M1) is built based on the observed crystallographic water molecules in the ba_3_ *T. thermophilus* X-ray structure of Tiefenbrunn et al. (Hunsicker-Wang et al., 2005; Tiefenbrunn et al., 2011) in the resting oxidized state (state O) and then geometry optimized using DFT in correspondence with the various forms of State P_R_ (see models a and b in Figure 8). Models c, d, e, and f are of state F-type, but with 2H_2_O and protons shifting positions from state to state. We put in the observed water molecules that directly H-bond to the various oxygen species, here the ${\text{Cu}}_{\text{B}}^{2+}$ –OH^−^ or ${\text{Cu}}_{\text{B}}^{2+}$ –H_2_O in the P_R_ → F → O_H_ sequence, the other nearby hydrogen bonding (hydrating water molecules) within the DNC active site, as well as the small water pool that is found at the top of the DNC, interacting with His376, Asp372, and Prop-A. Hydration models M2 and M3 have the positions of some water molecules significantly shifted, as we will describe later. All of these water molecules lie within the quantum cluster model. Later on, when we address the classical molecular dynamics (MD) studies, many additional water molecules will be included.

The DFT computational and structural model uses the OLYP-D3(BJ) exchange correlation potential with D3(BJ) for dispersion effects and an overall quantum cluster size of approximately 205 atoms for the DNC with small variations depending on the overall protonation state. Since, in contrast with the MD studies, the surrounding protein, including other redox centers, hydrating water molecules, and the membrane, are not included in the quantum chemical model, we employed a continuum dielectric with dielectric constant *ɛ* = 18.5 to represent the overall polar environment outside the quantum cluster model. When protons are added to the quantum cluster (labeled H^+^ uptake), the approximate Δ*G* calculations assume proton addition at pH = 7 from the donating dielectric medium. In the actual biological system, for the ba_3_ enzyme, the protons added to the DNC come from the K path and originate from a glutamic acid/glutamate, as mentioned previously. For proton transfers within the DNC, the Δ*G* values are calculated from the ΔpK_*a*_ values between the different groups, including the calculated zero point energy differences between residues or between residues and bound H_2_O or OH^−^.

Returning to the reaction pathway in Figure 8, state a, P_R_ (M1), corresponds to the state labeled P_R_ within the larger global reaction cycle in Figure 5. Proton uptake gives a DFT-calculated approximate Δ*G* = −8.8 kcal/mol for protonation of Tyr237^−^ to form neutral Tyr in state b. State b is also the same one where the p*K*_a_ (His376^+^) = 9.2, as mentioned above. The proton transfer from Tyr237 (neutral, protonated) to generate Cu^2+^–H_2_O from Cu^2+^–OH^−^ has a calculated Δ*G* = −2.7 kcal/mol. The following proton uptake at Tyr237 has a Δ*G* = −5.5 kcal/mol, see the angled arrow directed from state c → d. The water model is still in State M1 at this stage. For all the states described with hydration M1, we now focus on the water dimer, labeled (2H_2_O), which is closest to the OH^−^ or H_2_O, which is the Cu^2+^-bound ligand. In this dimer, the first H_2_O is directly H-bonded to the OH^−^ or H_2_O Cu^2+^ ligand, while the second H_2_O is H-bonded to the other water of the dimer [these two water molecules correspond to the crystallographic water molecules HOH604 and HOH608 depicted in Figure 3B]. We treat the whole water dimer (2H_2_O) as moving together to the upper water pool around His376 after the Tyr237^−^ is protonated. The calculated free energy cost is small, Δ*G*_wds_ = +4.2 kcal/mol for the process model d (M1) → model d (M2) (wds = water dimer shift). There is a major associated change in the histidine cation (His376^+^) p*K*_a_ from p*K*_a_(i) = 12.2 for (M1) before to p*K*_a_(f) = 6.5 for (M2) after the water dimer shift (i = initial, f = final), so $\Delta G_{\text{i}\to \text{f}}=-1.37\left(\text{p}K_{\text{a}}\left(\text{f}\right)-\text{p}K_{\text{a}}\left(\text{i}\right)\right)=+7.8$ kcal/mol for the relative Δ*G* change in proton binding. Deprotonation of the His376 cation has a far more favorable free energy change in the final state (water model M2) compared to the initial state (water model M1). The corresponding Δ*G*_deprot_ (His376^+^) = 1.37 (p*K*_a_(*f*) − *pH*) = −0.7 kcal/mol at pH = 7. Therefore, the total Δ*G* for state d (M1) → state e (M2) with a proton disassociated from His376^+^ is +3.5 kcal/mol, which is positive and small, followed by a small but negative proton transfer energy (−1.4 kcal/mol) from Tyr237 to restore the full protonation of the His376^+^ cation in state f. We attribute the large p*K*_a_ shift in His376^+^ to the descreening of the Cu^2+^–H_2_O charge distribution due to the water dimer relocation from the lower pool near Cu^2+^-H_2_O to the upper pool near His376^+^. We also separately tested the effect of adding two more water molecules to the upper pool while keeping the other water molecules in the M1 arrangement [(M1+2H_2_O) Model]. The calculated p*K*_a_ = 10.7 is just 1.5 pH units below the p*K*_a_ found in State d (M1). State e (M2) (p*K*_a_ = 6.5) is much different from either of these M1 states, supporting our descreening proposal. In State g, M3 has a similar hydration model to state M2, with the water dimer in the upper pool near His376^+^, but the H_2_O ligand to Cu^2+^ has moved to a hydrogen bonding orientation to the ferryl–oxo (FeO)(2+). In the next redox and proton uptake step, the active oxidized complex State O_H_ is formed with a bridging hydroxyl (OH^−^) derived from the oxo ligand to Fe. Considering the full catalytic reaction cycle, each full cycle generates 2H_2_O from [O_2_+ 4e^−^ + 4H^+^], as seen in Equation (1), and in Figure 5. These 2H_2_O products per cycle must flow out at some stage. We propose that these two water molecules per cycle become the H-bonding water dimer to the Cu^2+^–OH^−^ or H_2_O ligands, and subsequently the water dimer moves to the upper water pool before being expelled via the water exit pathways. A similar mechanism applies if the two water product molecules exchange with other water molecules in the reaction pathway before exit.

### 3.2 Proton loading network and transfers by DFT

**FIGURE 9:**
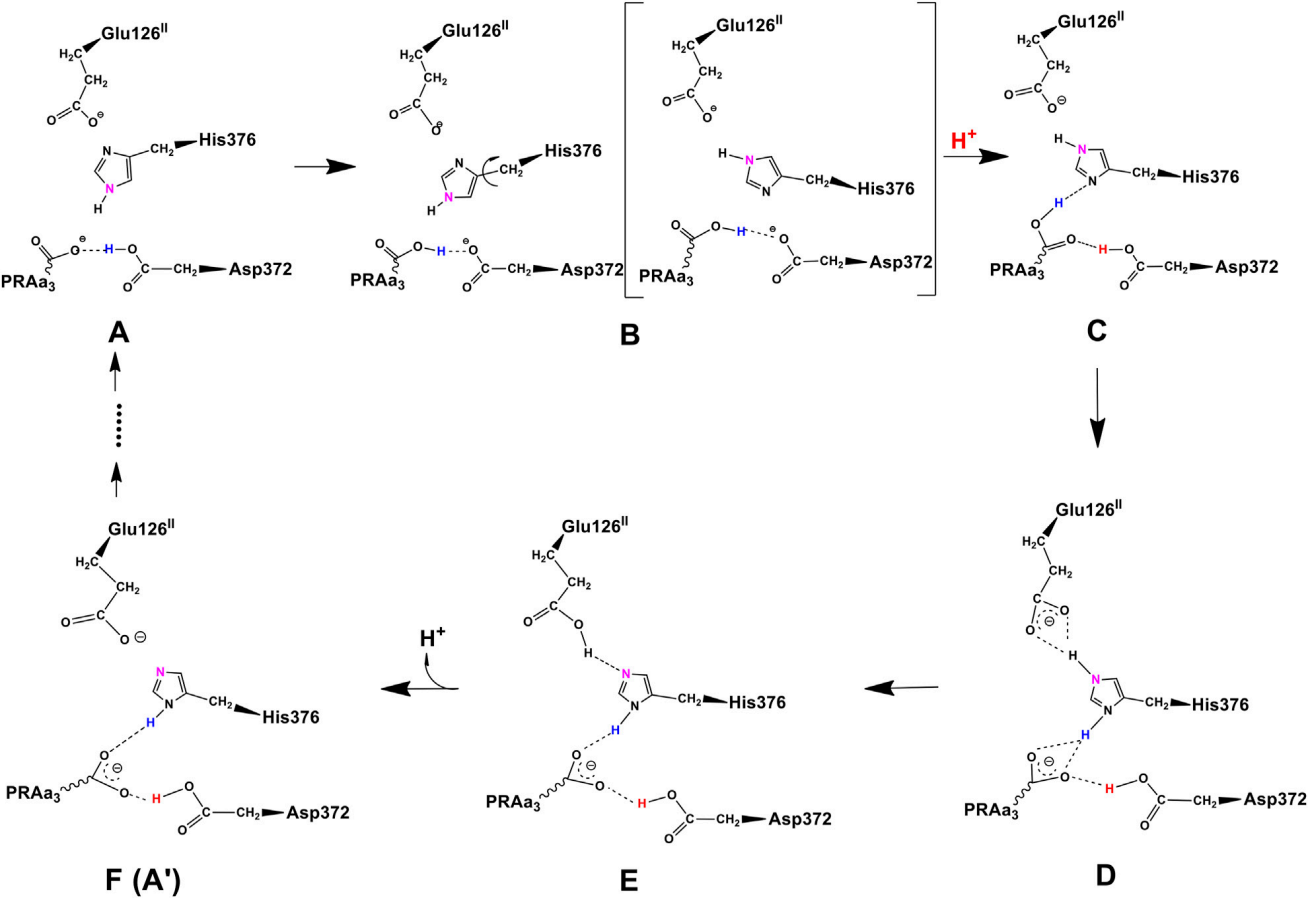
Feasible proton transfer pathways in the proton loading network (PLN) based on molecular dynamics simulations of the hydroperoxo state. Protonation state D is lowest in energy. Transferred protons and the *ɛ*-nitrogen on the imidazole ring of His376 are colored to guide the eye. **(A)** A strong hydrogen bond exists between protonated Asp372 and PRAa_3_, facilitating proton transfer. **(B)** Rotation of His376. **(C)** Proton uptake by Asp372 results in the formation of a strong hydrogen bond between protonated PRAa_3_ and His376. **(D)** Proton transfer from PRAa_3_ to His376 results in a strong hydrogen bond between His376 and Glu126^II^. **(E)** Proton transfer from doubly protonated His376 to Glu126^II^ and subsequent release into the water pool. **(F)** Structure and protonation state equivalent to **(A)** with the exception of the orientation of His376 and protonation site *δ*-nitrogen. Reproduced from Figure 1 in Yang et al. (2016a), copyright 2016 Elsevier.

**FIGURE 10:**
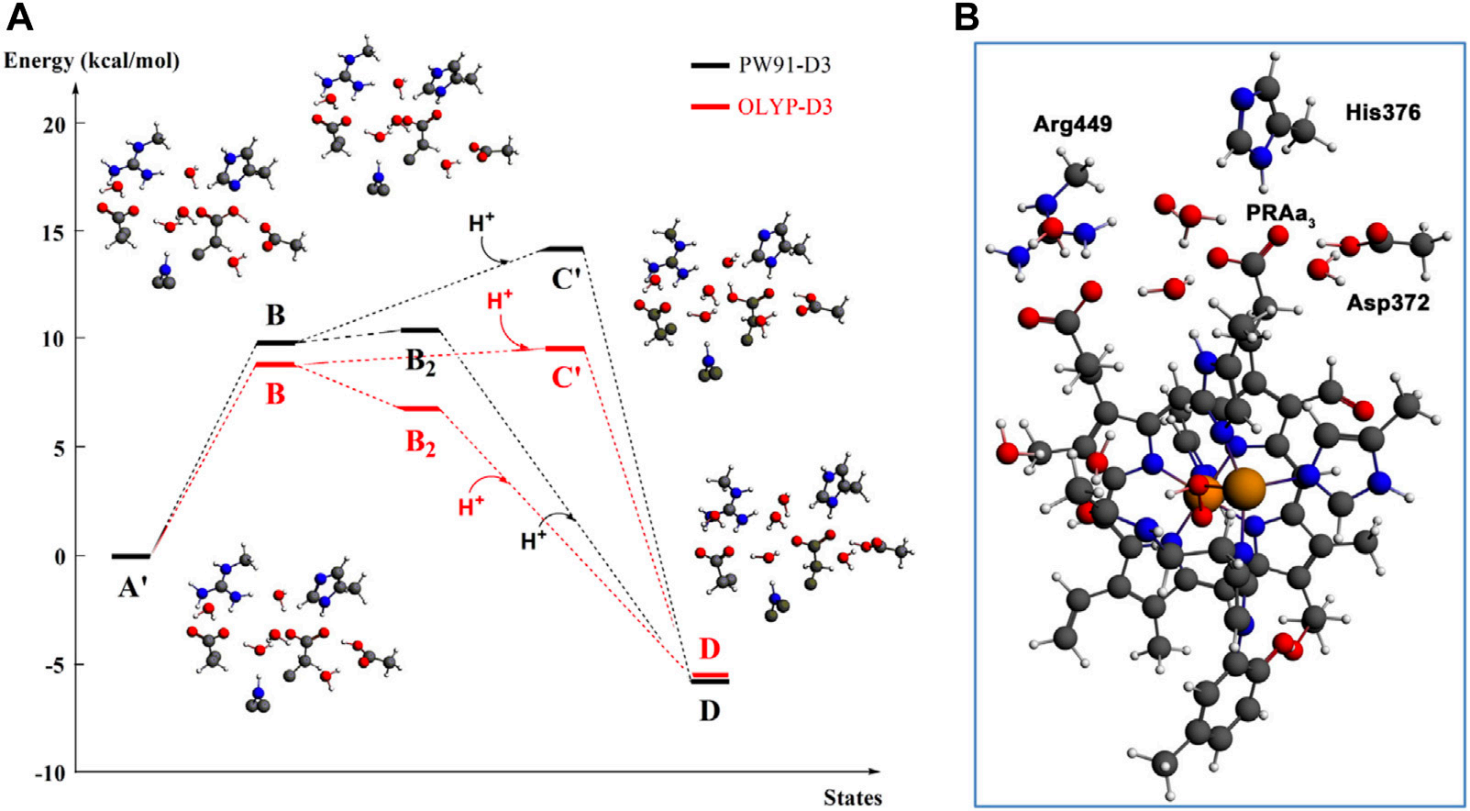
**(A)**: DFT energies for proton transfer and proton uptake from the N-side of the membrane (A’/B/B2 = cluster model + H^+^ at pH = 7) to His376 (D). The initial step is a proton transfer from Asp372 to PRAa_3_ (B). Protonation of His376 can occur either before proton uptake (B2) or after proton uptake (C′). The labels correspond to the nomenclature in Figure 9. The prime indicates *δ*-protonation in place of *ɛ*-protonation of His376. **(B)**: complete DNC cluster model for State A’. Reproduced from Figure 3 in Yang et al. (2016a), copyright 2016 Elsevier.

At this point, let us return to the problems of the proton uptake and proton transfer within the proton loading network (PLN) by quantum chemistry (DFT) and by molecular dynamics (MD) methods (Yang et al., 2016a). A schematic diagram for the various feasible protonation states involving the PLN is shown in Figure 9. All states of this proton uptake and proton transfer cycle have either two or three active protons, as found from our DFT calculations with OLYP-D3 (exchange-correlation potential with D3 dispersion correction). The PW91-D3 form of DFT gives generally similar relative energies. The DNC quantum cluster model we used in Yang et al. (2016a) corresponds to the hydroperoxo state in a model containing 204 atoms (205 atoms when there are three protons in the PLN). [We note that this hydroperoxo state has a free energy of protonation at pH = 7 for His376 → His376^+^ (−5.5 kcal/mol) similar to P_R_ state d (M1) (−7.1 kcal/mol) in Figure 8 and also has a hydration model like M1.] In the protonation and proton transfer cycle, state D, with His376^+^, is closely analogous to state d (M1); see below.) As shown in Figure 9, the main protonation sites in the PLN are on the Asp372 carboxylate sidechain, the proprionate A sidechain (PRA) of the heme a_3_, and the His376 ring nitrogen (both *ɛ* and *δ*). All of these groups are within the quantum chemical cluster model, and so the relative energies of these states can be calculated using DFT. We consider the sequence of states starting at state A (two protons in the PLN, on Asp372 and His376), and then a shift of one proton from Asp372 → PRA occurs with a histidine ring flip to form state B → proton uptake to form State C′, and another proton transfer from PRA → His376 to form His376^+^ (doubly protonated), State D. Figure 10 shows that the proton transfer from Asp372 → PRA is substantially endergonic (product State B) based on our DFT calculations both for OLYP-D3 and PW91-D3, and that state C′ lies well above state D with the same number of active protons (Noodleman et al., 2020) in this network. Asp372 is the most geometrically accessible protonation site from residues and water molecules below. As depicted in the figure, from state B → state B2 corresponds to a proton transfer from PRA → His376, which becomes (His376^+^, doubly protonated), and State B2 has a similar or lower energy than state B. State B2 also allows the proton uptake at Asp372 to result in the lower energy State D, while only the higher energy state C′ is directly attained by proton uptake from state B. Based on our earlier discussion, P_R_ state d (M1) → M2 will lead to much weaker proton binding in His376^+^ and readily allow the removal of a proton by Glu126 (subunit II) sidechain anion, as depicted in Figure 9, States E and then F (equivalent to State A′, His376 neutral, protonated at N *δ*). Furthermore, State C′ has a much higher energy than State D by 15 kcal/mol (OLYP-D3) or 20 kcal/mol (PW91-D3), while the p*K*_a_ of His376^+^ is near 11 or 12 in State D. This result implies that even after a strong lowering of this p*K*_a_ by approximately 6 pH units, backflow of a proton via State C (or C’) to the lower H-bonded network (vectorially toward the n-side) will be strongly diminished compared to the forward motion through state D → E → F and out through the residue sidechain-lined water pathways.

### 3.3 Proton loading and transfer by combining DFT and MD methods

By shifting the emphasis from the quantum DFT calculations to our classical molecular dynamics (MD) simulations [using the AMBER software package (Götz et al., 2012; Salomon-Ferrer et al., 2013a; Salomon-Ferrer et al., 2013b; Case et al., 2014) and AMBER ff12SB and Lipid14 force fields (Dickson et al., 2014; Maier et al., 2015)], we can see their implications. The details of the methods and the models for these MD calculations are presented in Yang et al. (2016a); Yang et al. (2016b), but a few main points should be emphasized. In states A, B, B2, C, and D, all the proton transfers lie within the overall quantum cluster. In States E, F, and A, however, proton transfers occur outside the quantum cluster region. Therefore, to analyze all of these states, MD models include the whole protein along with the metal cofactors (Cu_A_ cluster, heme b Fe, and the dinuclear heme a_3_ Fe—Cu_B_), as well as a representative membrane, explicit solvent and many mobile water molecules in the protein interior, and ions for overall neutrality. The partial charges of the metal cofactor regions are calculated from DFT (OLYP-D3) to obtain electrostatic potential (ESP)-based charge distributions which are combined with standard charges from an AMBER force field model and other AMBER force field parameters. The protonation states of the various protein sidechains and for proprionate A are given in Table 1 of Yang et al. (2016a), and the fractional hydrogen bond occupancies for the different states from the MD simulations in Table 2 and the Supporting Material there (states A′, B′, and C′ are alternate tautomers to States A, B, and C (protonated at N *ɛ*) on His376 (neutral). These alternate tautomers are expected to have very similar energies. During these dynamics simulations, in addition to fluctuating hydrogen bond linkages and water mobility, ring flips of His376 and movements of nearby sidechains are frequent for simulations from any of the starting States A–F (or States A’, B’, and C’).

**FIGURE 11:**
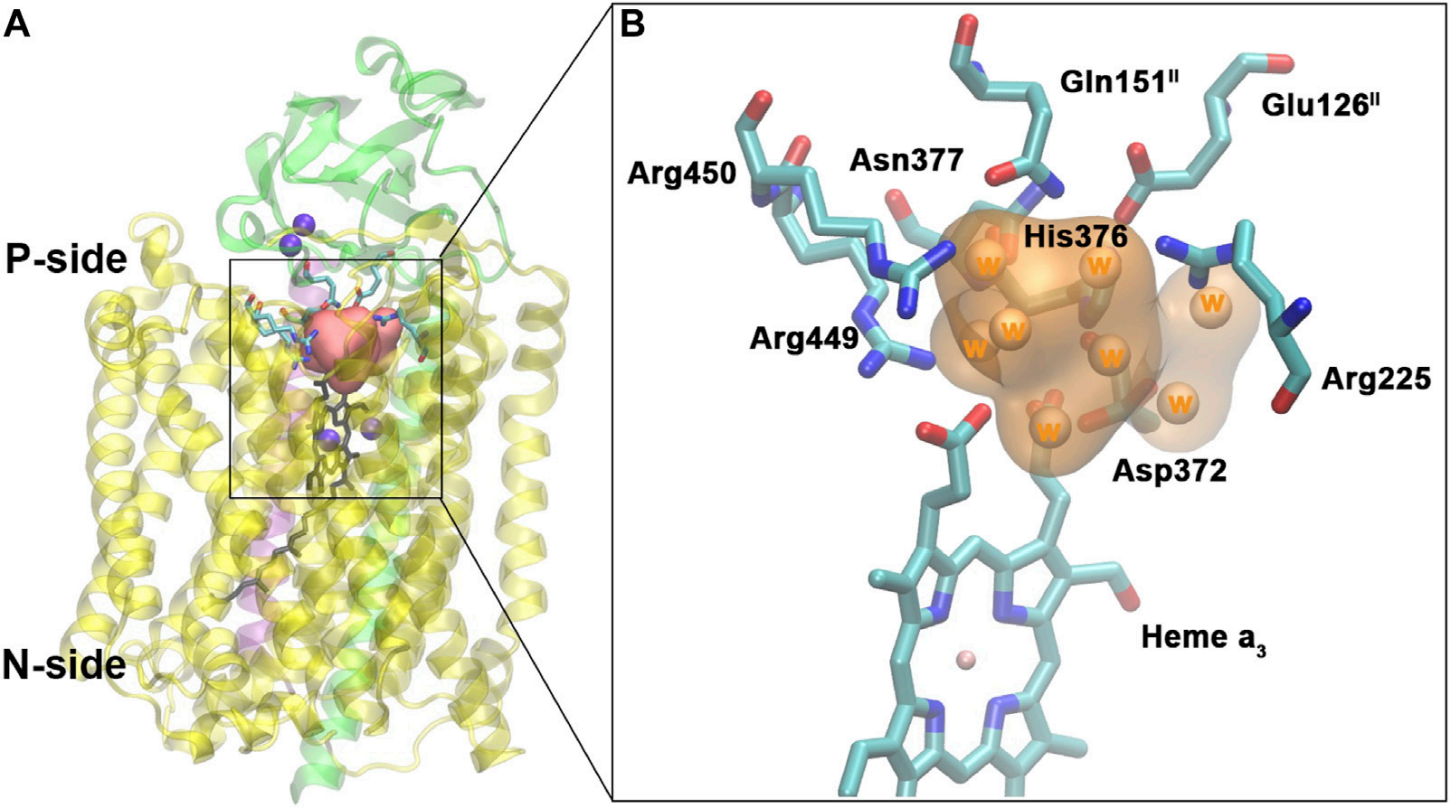
Water pool above the DNC in the crystal structure of ba_3_-type CcO from *Thermus thermophilus* (PDB ID 3S8F). **(A)**: Location of the water pool within the enzyme with surrounding residues highlighted. Purple spheres are metals in the protein (Cu_A_, Cu_B_, and Fe_a3_). Subunits I, II, and III are shown in yellow, green, and purple, respectively. **(B)**: Details of the water pool and surrounding residues. The orange region is the water pool consisting of eight crystal water molecules. Reproduced from Figure 5 in Yang et al. (2016a), copyright 2016 Elsevier.

**FIGURE 12:**
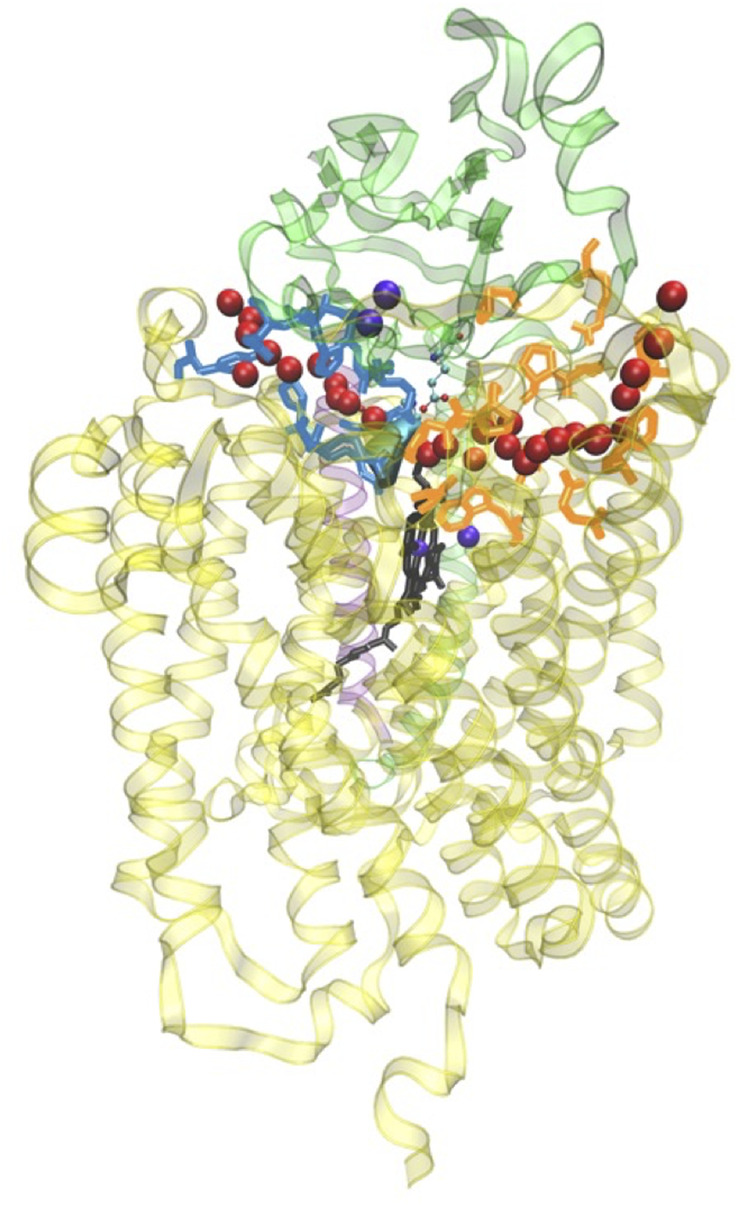
General view of the two water exit pathways that connect the water pool above the DNC to the P-side of the membrane. Residues in blue color line pathway P1, and residues in orange color line pathway P2. The positions of water molecules along one branch of each of the pathways are depicted with red spheres. Reproduced from Figure 6 in Yang et al. (2016a), copyright 2016 Elsevier.

**FIGURE 13:**
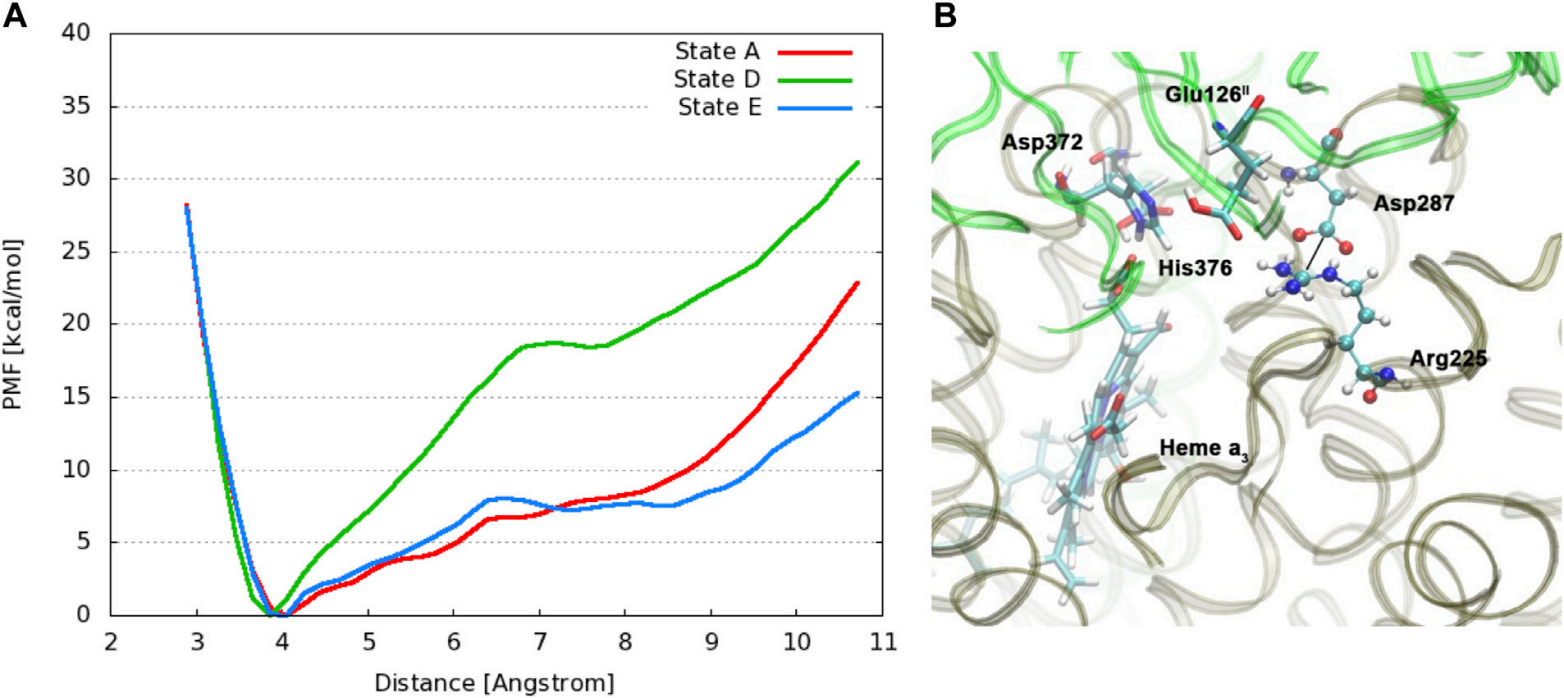
**(A)**: Free energy profiles for the salt bridge Arg225–Asp287 in different protonation states of CcO. The distance is measured between the carbon atom C_*ζ*_ of the guanidinium group in Arg225 and the carbon atom C_*γ*_ of the carboxylate group in Asp287. (Dotted line) **(B)**: Snapshot from a simulation of state E with protonated Glu126^II^, highlighting relevant residues. Reproduced from Figure 4 in ref. Yang et al. (2016a), copyright 2016 Elsevier.

Changing the perspective from proton transfer energies, we now consider changes in geometries with related energies, water mobility, and occupancies as described by MD methods. Over a selected finite simulation time (140 ns), we have calculated the number of H_2_O molecules that exchange from the upper water pool to bulk water or vice versa via two pathways (small water channels), P1 and P2. The upper water pool is bounded by His376, Asp372, PRA carboxylate, and other adjacent residues, sidechains of Glu126^II^, Gln151^II^, Arg225, Asp287, Arg449, Arg450, and Asn377. See Figures 11, 12. We do not count the water molecules within these two channels. The total number of H_2_O molecules exchanged (total for P1 + P2) for States A, B, C, D, E, and F (A’) are 15, 2, 5, 0, 5, and 16. Clearly, state D shows the least exchange and states A and F the most exchange. The proton released from state D (His376^+^) to Glu126^II^ and then onward opens these water exchange channels. We can expect that when the His376^+^ p*K*_a_ value is greatly lowered, the proton transfer from His376^+^ to the Glu126^II^ anion will eliminate that salt bridge, which then leads to H_2_O exchanges. Another intact salt bridge, Arg225–Asp287, is observed nearby. We have calculated the potential of mean force (PMF using MD) by displacement of the reaction coordinate between C(*ζ*) of the guanidinium from Arg225 and carbon C(*γ*) of Asp287 carboxylate, Figure 13. This salt bridge does not break, but the distortion to larger distances costs far more free energy in State D than in States E or A. The resistance to stretching the Arg225–Asp287 salt bridge is also consistent with the very low water mobility we find between the upper water pool and bulk water in State D. Overall, the change in the electrostatic potential within the DNC, particularly near the ${\text{Cu}}_{\text{B}}^{2+}$ site with a water shift, promotes the water flow and proton exit from the upper water pool through the two exit channels.

### 3.4 Splitting the proton entry path into inner and outer branches

**FIGURE 14:**
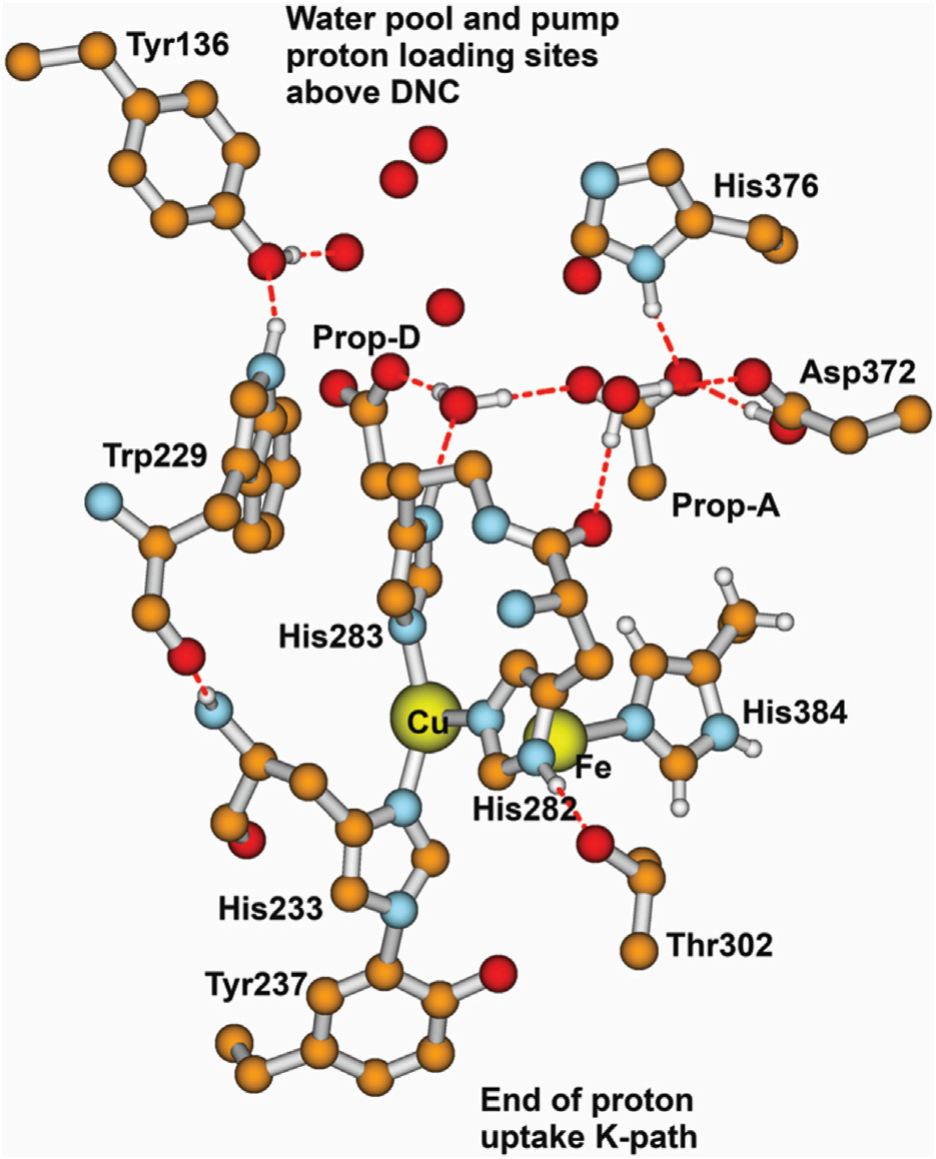
Major residues and water molecules in the DNC of the Tt ba_3_ X-ray crystal structure 3S8G. For clarity, only Prop-A and Prop-D of the heme ring are presented here; the whole heme ring is not shown. Reproduced with permission from Figure 15 of Noodleman et al. (2020).

Another large-scale problem arises, i.e., where does the proton entry path split into two, with one path (the inner path) entering the Cu_B_–Fe_a3_ “reaction chamber” (language from JA Fee), and the other path (the outer path) providing vector protons for binding to the proton loading network and exit? How are these two paths sequenced or synchronized? It is clear that Tyr237 is involved at the end of the proton uptake K path, but what occurs after that point? Figure 14 depicts that structural region in a different way from that in earlier figures, emphasizing the surroundings of Cu_B_. For the inner path, let us concentrate on the pathway for forming the ${\text{Cu}}_{\text{B}}^{2+}$ bound hydroperoxide starting from the reduced state R, which, upon binding O_2_, becomes a ferric–superoxide complex (Fe^3+^– ${\text{O}}_{2}^{-}$ ) (state R → state A → hydroperoxide). We propose this negative charge transfer from Fe to O_2_ triggers strong geometric changes and a proton transfer involving Cu_B_, which is in the cuprous Cu^+^ state. Cu^+^–histidine(N) bonds can be labile, and then the broken bond can protonate at the histidine nitrogen. Starting from Figure 14, we propose that His283, during Cu^+^–His(N) cleavage, picks up a proton by a ring flip from His282 protonating to His283^+^, and this proton loss on His282^−^ is then restored by Thr302, whose proton is resupplied by Tyr237 and other amino acid carriers and water molecules. Both the local Cu ligands and Thr302 (or similarly a serine) are well-conserved isostructurally across ba_3_ and aa_3_ CcO′s. Trp229 is also structurally well-conserved and well-placed to form a stabilizing *π*–cation interaction with His283^+^. From this point, a 1e^−^ transfer from Cu^+^ to the ${\text{O}}_{2}^{-}$ will be linked to a proton transfer from His283 to form the hydroperoxide state, which will also allow for reformation of the Cu^2+^–His(N) covalent bond. This proton transfer is likely concerted with the 1e^−^ transfer, although these may be asynchronous. This pathway avoids two problems: (Rouault and Tong, 2008) direct electron transfer from Cu^+^ to ${\text{O}}_{2}^{-}$ and bond formation without a rapid proton transfer could yield the very-low-energy peroxo state Fe^3+^–(O–O)^2−^–Cu^2+^ (DFT OLYP-D3BJ energy calculated as 12 kcal/mol below the following hydroperoxide state, protonated near Cu^2+^), which would strongly inhibit proton transfer from Tyr237 according to our DFT OLYP-D3-BJ calculations (Han Du et al., 2019). In this same work, we noted that the peroxo bridge structure is considerably distorted geometrically compared to the superoxide state (state A) with a peroxo calculated Fe–Cu distance of 3.88 Å and Cu–O distance of 1.94 Å, compared to Fe–Cu 4.55 Å, and Cu–O distances between 2.95 and 3.2 Å in state A. From a kinetics viewpoint, coordinating large charge transfers and large geometry changes likely requires a high reaction barrier. By contrast, the hydroperoxo state has a bridge structure, calculated Fe–Cu distance 4.31 Å, and Cu–O distance 2.11 Å, closer to these distances in state A (Nicholls and Ferguson, 2002). The alternative proton pathway from Tyr237 to a nearby water molecule and then into the peroxide-bound state is long (approximately 4 Å) and likely results in a proton bound to the peroxide, generating a hydroperoxide at the higher-energy Fe^3+^ side instead of placing the OH near Cu^2+^ (OLYP-D3 energy difference 11.8 kcal/mol) (Han Du et al., 2016). By contrast, the mechanism we propose is consistent with the mobility and linkage of proton transfers seen in other systems (Grotthuss process) (de Grotthuss, 1806) and the lability of Cu^+^–histidine bonds (See Table 2).

In earlier work, we proposed a similar mechanism from DFT calculations and found strong supporting evidence from the X-ray structure in the reaction pathway of Cu–Zn superoxide dismutase (CuZnSOD) (Konecny et al., 1999; Shin et al., 2009; Perry et al., 2010).

Here, the first 1e^−^ transfer from the initial superoxide bound to Cu^2+^ forms Cu^+^, and O_2_ leaves. Then, a following 1H^+^ transfer breaks a Cu–histidine bond (to the histidine bridging Cu to Zn covalently). When a second superoxide enters and reacts with Cu^+^, the 1e^−^ transfer to ${\text{O}}_{2}^{-}$ is accompanied by deprotonation of that histidine, formation of hydroperoxide (OOH)^−^, and reformation of the Cu^2+^–His bridge bond to Zn^2+^. X-ray structures of CuZnSOD in the reduced Cu^+^ form have a broken Cu–His bond, while in other structures of oxidized proteins, this bond is intact.

In answer to the initial question raised, it is likely that Thr302 (or similar isostructural Thr or Ser in other CcO′s) is a significant branch point: a negative local electrostatic potential in the (Fe^3+^– ${\text{O}}_{2}^{-}$ )–Cu^+^ state A drives protonation at His283 near Cu^+^ in the inner path, while in the outer path, a positive electrostatic potential near Cu^2+^–H_2_O and Tyr-OH drives deprotonation of His376^+^ and proton recovery (from State B2) via proton transit from Thr302 to Asp372 using intervening water molecules and amino acid sidechains. As discussed before, State C’ lies well above State D by 15–20 kcal/mol. Using a simple Marcus-type model for the reorganization energy and driving force associated with the proton transfer from state A’ (reactant) to state C’ (product) *versus* state A’ to state D (product) to lowest order (linear), the ΔΔ*G* (activation) = Δ*G* (C’ → D)/2, so the activation barrier is calculated to be lower by 7.5–10 kcal/mol in State D compared to State C’ (see chapters 5 and 7 in Connors (1990).

After O–O bond cleavage, the formation of the radical transient state P_M_ is expected with a Tyr237 radical (ba_3_) until 1e^−^ is transferred from the mononuclear low-spin heme b (in ba_3_) or analogously mononuclear low-spin heme a (in aa_3_ enzymes). During normal cycling, the electron transfer from heme b or heme a is rapid. In contrast, Yu et al. (2012) trapped two different Tyr radicals with their spectra determined by X-band and D-band EPR and further analyzed with QM-MM methods to compare calculated spin distributions with ring hyperfine parameters. These radicals were trapped at a low concentration in bovine CcO (aa_3_-type). A mixed valence state that stops at P_M_ was obtained by starting with the oxidized bovine CcO and reacting this with hydrogen peroxide (HOOH). (Hydrogen peroxide does not have access to the active site in ba_3_ CcO as in *Thermus thermophilus* in the oxidized state, so the analogous experiment has not been done.) These two Tyr radicals in bovine aa_3_ CcO are Tyr244, covalently linked to His240, and Tyr129, with a nearby Trp236 which are analogous to Tyr237 linked to His233, and Tyr136 near to Trp229 in ba_3_ CcO (*Thermus thermophilus*). Figure 14 shows the Cu_B_ near surroundings based on the Tt ba_3_ X-ray crystal structure. The covalent linkage of Tyr237 to His233 is shown. The His233 main-chain amide is hydrogen bonded to the Trp229 main-chain carbonyl. It is also evident from the residue numbering that there is a covalent linkage as well between His233 and Trp229 by a longer pathway. Yu et al. have proposed (see their Figure 7) that at P_M_, a radical transfer mechanism can move a hole from the special Tyr244 radical to the higher positioned Tyr129, forming a cation radical with a loss of a proton later from the same tyrosine (Yu et al., 2012). Hole motion from Tyr244 to Tyr129 is equivalent to 1e^−^ transfer from Tyr129 to Tyr244, forming a Tyr244^−^ anion. The proton on Tyr129^+^ cation radical will exit via the D-path available in bovine aa_3_ CcO, and after Tyr129 is 1e^−^ reduced by the mononuclear heme a, another proton will bind to Tyr129 from another position of H-bonded groups lower along the D-path. Something similar appears likely to us in ba_3_-type CcO. We emphasize more the role of the intervening Trp229 in Tt ba_3_ (see Figure 14) (Noodleman et al., 2020). After the formation of the Trp229^+^ radical cation state, the nearby Tyr136 sidechain will be deprotonated via electrostatic repulsion. The Tyr136^−^ anion formed may persist or may exchange an electron with the Trp229^+^ cation radical. In either case, the Trp229^+^ radical or the Tyr136 neutral radical then receives an electron from the Fe^2+^ on heme b. The Tyr136^−^ anion formed has its proton restored from His376 and adjacent residue sidechains, which are nearby.

Toward the end of the oxidative cycle, we see the transition from the state labeled (f,M2) → (g,M3) → (h) (State O_H_) (Figure 8). The first two states named are different forms of state F, where the H_2_O ligand on Cu^2+^ shifts to a hydrogen bonding position to the ferryl–oxo oxygen (Fe^4+^ = O), which is then followed by a high redox potential coupled electron–proton transfer (e^−^,H^+^), calculated by DFT to have a Δ*G* = −9.5 kcal/mol, calculated with respect to a typical cytochrome c redox potential (approximately 0.22 V *versus* SHE). This is one of the slower transitions involving protonation within the reaction chamber, here of an oxo to hydroxo state, along with the electron transfer. We propose a feasible mechanism for this reaction. In this mechanism, the H-bonded water transfers one proton to the oxo after or concerted with the one-electron transfer to the Fe^4+^ → Fe^3+^. The newly formed hydroxyl (the one not bound to Fe^3+^) will then first activate protonation of the Tyr237^−^ anion and then proton transfer to that hydroxyl to re-form an H-bonded water. This looks to us like the most probable mechanism. In state (g,M3), the DFT-calculated p*K*_a_ is quite low, p*K*_a_ = 1.8 to 4.0, but a nearby nearly free OH^−^ should increase the effective p*K*_a_ of Tyr237 via electrostatic stabilization for subsequent proton transfer to the same OH^−^. Over the full reaction cycle, the mechanisms for state A → hydroperoxo and state F → state O_H_ that we have proposed account for the entry of two (out of four) chemical protons into the reaction chamber to bond to various oxygen species (see Table 1). The coupled (1e^−^,1H^+^) transition from State O_H_ → E_H_ reduces Cu^2+^ to Cu^+^, so a mechanism similar to the one proposed for state A → hydroperoxo is likely feasible. The mechanism for the entry of the chemical proton to form Cu^2+^–H_2_O from Cu^2+^–OH^−^ (P_R_ → F) in Figure 5, or in more detail, state (b,M1) → state (c,M1) in Figure 8 is not clear, although the DFT-calculated Δ*G* = −3 kcal/mol approximately. We have also shown results from DFT and MD calculations supporting a mechanism for pumping of one vector proton in the state P_R_ → state F transition. For the preceding State P_M_ → state P_R_ transition, we think a mechanism similar to the one proposed by Rousseau et al. (Yu et al., 2012) for aa_3_-type CcO based on EPR experiments applies also to ba_3_ CcO in *Thermus thermophilus*.

### 3.5 Aspects of DFT parameters employed in our work

In terms of DFT exchange-correlation potentials, generalized gradient approximations (GGA) (including OLYP and PW91) are much more computationally efficient than hybrid DFT–Hartree–Fock methods (like B3LYP and B3LYP*), particularly when optimizing geometries. We have calculated geometries for some families of Fe complexes using OLYP and PW91, particularly iron–sulfur, iron–sulfur–nitrosyl, and iron–carboxylate–nitrosyl systems, and found that the predicted geometries agree better with the experiment than B3LYP or B3LYP*.

For relative spin state energetics, Vancoillie et al. (2010) have found that OLYP yields similar good-quality results with respect to experiment when compared to B3LYP and B3LYP*. Currently, all practical methods for predicting relative spin state energetics for Fe complexes and other transition metal complexes are not completely reliable, but some perform well on many systems. Radoń and Pierloot (2008) compared high-level CASSCF/CASPT2 calculations to PBE0, B3LYP (hybrid DFT–Hartree–Fock methods), and to OLYP, BP86 (GGA methods) (note: BP86 is similar in behavior to PW91) for binding energies of CO, NO, and O_2_ to two model heme systems. OLYP and BP86 best reproduced the binding energies of CASSCF/CASPT2. Potentials BP86 and PW91 often give good binding energies, but over-stabilize low-spin states for Fe and other transition metal complexes, while OLYP and OPBE are more balanced in providing accurate relative spin state energies. Since the reaction cycle for cytochrome c oxidase involves both oxygen species binding, Fe and Cu spin state changes, redox changes, and protonation/deprotonation, we have emphasized OLYP in our more recent work published in 2014 and later.

D3 force field-type corrections for dispersion effects became available (from Grimme et al.), and we utilized these to further improve geometries in our work combining DFT and molecular dynamics studies (OLYP-D3, PW91-D3) (Yang et al., 2016a) and in our prior Fe–Cu hydroperoxide cleavage work (Han Du et al., 2016). Further improvements to dispersion force field terms yielded the D3(BJ) term, which was incorporated to obtain results with OLYP-D3(BJ) and PW91-D3(BJ) in our papers in 2018 (Han Du et al., 2018) and later (Han Du et al., 2019). Some B3LYP-D3(BJ) single-point calculations using PW91-D3(BJ) optimized geometries were also done.

DFT basis sets are Slater-type using the ADF code (te Velde et al., 2001; van Lenthe and Baerends, 2003; Baerends et al., 2017), triple zeta with polarization functions (TZP) for Fe and Cu, and double-zeta with polarization functions (DZP) for all other atoms, with inner core orbitals frozen. Basis sets do vary based on the requirements of the properties being calculated, for example, Mossbauer properties (no frozen core on Fe, Cu) or vibrational frequencies, where we have sometimes expanded the basis set.

### 3.6 Additional connections to the literature on cytochrome ba_3_ and aa_3_

For a very recent review from a broader viewpoint, we recommend the paper of Wikstrom, Gennis, and Rich, which combines an analysis of structures, kinetics, and spectroscopy with some theory/computation as well (Wikström et al., 2023). For DFT calculations and reaction energies for the aa_3_ chemical cycle, the recent paper of Blomberg (2021) is valuable as it shows a different approach from ours.

A recent study of the reaction kinetics of ba_3_-type CcO in Tt at a low temperature (10 C) clearly shows the presence of an additional intermediate state just after the hydroperoxo state (see Figure 5), but before the O–O cleavage product state P_R_ (Poiana et al., 2017). In the fully reduced enzyme, after O_2_ binding, optical difference spectroscopy shows that beginning at State A, one electron leaves the heme b Fe^2+^ well before the O–O cleaved P_R_ state is observed. The relevant time constant ratio is 1/10 for the oxidation of heme b compared to the formation of P_R_ at 10 C, but near 1/1 for this ratio at 45 C, which is closer to the typical physiological temperature range for *Thermus thermophilus* species (50 C–80 C in hot springs) (Dyer, 2003). These results implicate a newly observed low-lying intermediate state at the dinuclear reaction site, presumably with Cu_B_ again reduced to Cu^+^. We have proposed that in forming the hydroperoxo state, there is initially a broken bond between His283^+^ and ${\text{Cu}}_{\text{B}}^{+}$ before (1e^−^,1H^+^) transfer to the Fe^3+^-superoxide state. If after these transfers, the re-formation of the His283 to ${\text{Cu}}_{\text{B}}^{2+}$ bond is slow, the reduction to ${\text{Cu}}_{\text{B}}^{+}$ may occur with a small reorganization energy. In addition, this last 1e^−^ transfer from heme b Fe^2+^ reducing Cu_B_ to Cu^+^ can potentially occur either as a single-electron tunneling event or alternatively with a hole-type (amino acid radical) intermediate along the pathway.

There are very significant differences between the proposed aa_3_ CcO pathways and what we think are feasible and probable in ba_3_ class CcOs. In particular, aa_3_ CcOs have two proton input paths K and D. Most proton transfers occur via the D-path, see Table 1. Along the D-path, there are water molecules and H-bonded residues where protons, one at a time, can be diverted by a glutamic acid in some proposed mechanisms toward a proposed proton loading site, suggested to be His291 and/or the nearby proprionate D, which can, in principle, be part of the chemical proton transfer path (His291) or part of the proton pumping path (His291 or Prop-D) (Popović and Stuchebrukhov, 2004; Popović, 2013). Geometrically, the His291 is above the Cu_B_ and near a small water pool. The combined DFT/electrostatic analysis (Popović et al., 2005; Popović and Stuchebrukhov, 2005; Makhov et al., 2006; Blomberg and Siegbahn, 2012) supports the presence of His291 as an anion (His291^−^), which can then be one site for proton loading, neutralizing the His291 charge, to be further transferred to oxygen species, like OH^−^, or pumped out. A number of other proton loading and exit pathways have been proposed in aa_3_ CcOs, including sites near Prop-D, near Prop-A, or near the low-spin heme a, triggered by electron transfer from heme a Fe^2+^ to heme a_3_ Fe^3+^ or Fe^4+^. In this context, early papers by Blomberg et al. (2000); Siegbahn et al. (2003) used DFT calculations on aa_3_ from bovine heart mitochondria to calculate the O–O bond cleavage pathway, redox potentials, and p*K*_a_ values for metal-bound groups and other groups, including tyrosine. A later paper on aa_3_ class CcO in bacterial *Rhodobacter sphaeroides* used a larger quantum model (approximately 250 atoms) to focus on proton transfer near Prop-A and Prop-D of heme a (Blomberg and Siegbahn, 2010).

In ba_3_ class CcOs, there is only one proton input path, the K path, which is located in a similar position to the K path in aa_3_ CcOs (see Figure 4) but has some differences in the amino acid and water molecule placements. In any event, the absence of a D-path in ba_3_ class CcO (Tt) and the resulting use of the K path for both chemical and vector (pumping) proton pathways leads to different mechanisms. The Cu_B_ ligands in mitochondrial CcO are His240, crosslinked to Tyr244, His290, H-bonded to Thr309, and His291. The corresponding Cu_B_ ligands in Tt CcO are His233 (crosslinked to Tyr237), His282, H-bonded to Thr302, and His283 (in a similar structural position to His291). We note that this Thr or Ser is broadly conserved in CcOs, both aa_3_ and ba_3_. With protons coming from the K path in ba_3_ CcO, our proposed mechanism for protonation of superoxide, ${\text{O}}_{2}^{-}$ radical (state A) involves a His283^+^ cation which dissociates from Cu_B_ in the Cu^+^ state, a transient His282^−^ anion, and reprotonation from Thr302 via Tyr237 and the K path. We think that Thr302 is also a branch point for proton transfer via water molecules to Asp372. Therefore, these mechanisms are quite different, including which histidines are charged and when, but so are the proton input paths in aa_3_ *versus* ba_3_ enzymes.

For state P_R_ and state O_H_ in ba_3_, we have calculated p*K*_a_ values and the redox potentials for Tyr237. These results are also relevant for vibrational frequency and pKa measurements for other tyrosines Tyr136 and Tyr133. For the P_R_ state, the p*K*_a_ (H_2_O) for H_2_O/OH^−^ bound to ${\text{Cu}}_{\text{B}}^{2+}$ with TyrO^−^ equals 15.4. The p*K*_a_ (Tyr) for TyrOH/TyrO^−^ with OH^−^ bound to ${\text{Cu}}_{\text{B}}^{2+}$ equals 13.4. The Δ*G* for proton transfer from TyrOH to ${\text{Cu}}_{\text{B}}^{2+}$ OH^−^ is thus −2.7 kcal/mol. The calculated redox potential for TyrO^−^/TyrO^⋅^ with respect to SHE is +0.05 V. The calculated redox potential for TyrOH/TyrO^⋅^ with respect to SHE is +0.43 V, which is considerably higher, as expected, and also higher than that of cytochrome c. pH = 7 is assumed for the protonation state of the surrounding medium acting as the proton source. The cytochrome c redox potential is set empirically to +0.22 V. The Tyr is Tyr237 covalently linked to His233. Broadly, our results and proposed models agree with spectroscopic studies by Koutsoupakis et al. (2011) since the Tyr237 often has a high p*K*_a_, while they observe moderate p*K*_a_ near 7.8 for other Tyr, presumably Tyr136 and/or Tyr133, through different reaction cycle states, showing shifts in the intensities of vibrational frequencies reflecting protonated *versus* deprotonated tyrosines. Tyr136 is along one exit path in our MD studies (Yang et al., 2016a). Tyr136 also plays a role in our proposed radical transfer mechanism in ba_3_, which follows the original experimental EPR work of Rousseau et al. on aa_3_. These data come from our recent papers (Han Du et al., 2018; Han Du et al., 2019).

For state O_H_, by comparing the OH^−^ symmetric bridge to the H_2_O bridge in different geometries, we calculated the bridge H_2_O p*K*_a_ between 11.7 and 12.2 (22). We did not calculate the corresponding TyrOH to TyrO^−^ p*K*_a_. Following the trend of prior states in the reaction cycle, we assumed that Tyr237 is deprotonated here (TyrO^−^) and then would pick up protons during the reductive part of the reaction cycle.

Cai et al. use electrostatic and molecular dynamics methods to characterize the protonation states and their statistical occupancies including relative energies during proton exit when stepping through the various redox, protonation, and oxygen species binding in the chemical cycle (Cai et al., 2018; Cai et al., 2020). The chemical cycle states interact via electrostatics with the protonation states along the exit path. There is no quantum chemical treatment of the catalytic reaction cycle, so that part of the energy is omitted. For the ba_3_ enzyme in CcO, the authors focus particularly on the sites Asp372, Prop-A, His376, and Glu126(II). They find, on average, 1–2 “active protons” on these sites. This agrees with our count of 2–3 protons in this region before proton release with water outflow. There is a difference in language here since they count His^+^ as having one “active” proton, while we count two protons (total) on His^+^. More significantly, in our work, we find that Prop-A when protonated is in a high-energy state, which helps block proton backflow. Cai et al. find some states where Prop-A is protonated at low energy, which disagrees with our results.

For the combined water–proton exit paths, there is a body of work dealing with the coordination geometry and energies of protonated water clusters, particularly the ${\text{H}}_{5}{\text{O}}_{2}^{+}$ Zundel-type ion bonded to anionic amino acid sidechains, as found in the electrostatics modeling of bacteriorhodopsin (Spassov et al., 2001). This is one example of a class of related possibilities for proton–water exit mechanisms (see Table 2, last three lines). Next, we discuss aspects of the sequence and structure dependence of proton–water exit pathways in ba_3_ *versus* aa_3_.

In a publication by Sugitani and Stuchebrukhov (2009), the proposed exit pathway of aa_3_ and ba_3_ class enzymes is compared, using the different ba_3_ enzyme structure to propose a different exit pathway than in the aa_3_ enzyme. Their proposed ba_3_ pathway agrees very well with our proposed pathway, called P1, see Figure 12. See also Supplementary Figure S5 in the Supplemental Material of our work, where we find two pathways P1 and P2, as shown in the figures in Yang et al. (2016a).

Gennis et al. studied reaction cycling and proton pumping via related exit pathways for various site-directed mutants in the exit pathways (Chang et al., 2012). There is no clear explanation for the specific effects of these different site-directed mutations on pumping and on catalytic cycling. We can make a few general comments based on our work, but we have analyzed only the native state of ba_3_ CcO from *Thermus thermophilus*, and not from the mutants. Their experimental observations are that some point mutations of His376 and Asp372 keep proton pumping intact (H376Y, H376F, D372N, and D372V), while a few others disrupt proton pumping (H376N and D372I). In addition, other mutations produce low electron turnover in the catalytic cycle (H376A, H376D, and D372E), so residues in the exit path can disrupt the chemical catalytic cycle as well. Based on these experiments, our view is that there are multiple solutions to these exit paths. The proton pumping mechanism we have proposed for the transition of state P_R_ → state F is connected to a large shift in the p*K*_a_ of His376 by long-range electrostatic effects. Similar large shifts may be feasible for other residues substituting for His376 or nearby residues. Furthermore, the “typical” p*K*_a_ values of residue sidechains can vary substantially depending on the surroundings, and even single-point mutations can alter local geometries and nearby water clusters. In addition, we find that Prop-A facilitates proton exit by blocking proton backflow, an effect which could well-persist after mutations near Prop-A. In addition, our proposed radical transfer mechanism in state P_M_ leads to deprotonation of Tyr136 at a different location along the water and proton exit path. However, these comments do not answer the detailed issues raised by the proton pumping experiments in Chang et al. (2012).

In a very recent work, Yang et al. (2023) have analyzed the effects of some of these site-directed mutants of ba_3_ CcO on the proton exit pathways using electrostatics and MD methods closely related to those we used in our collaborative work on the native enzyme (Yang et al., 2016a). The authors find that often site-directed mutations change structures of adjacent residue sidechains and that the water pathways are shifted often, consistent with our speculations mentioned above. They have been able to rationalize some experimentally observed effects of these mutants on proton exit, but many open questions remain.

## 4 Conclusion

We briefly summarize some of the main conclusions from this work.

1) DFT calculations find structures and vibrational frequencies along a reaction pathway that closely correlate with Fe–O frequencies observed by resonance Raman spectroscopy. Calculated Fe–O and O–O Mayer bond orders provide further insights into bonding and associated changes in geometries.
2) We develop the water shift mechanism for proton pumping by combining DFT and molecular dynamics work. Two water molecules per cycle are reaction products, but these two water molecules (or equivalently other water molecules that exchange with them) shift their positions when both Cu(II)–OH^−^ and Tyr(O)^−^ within the dinuclear center (DNC) are protonated, descreening and affecting histidine deprotonation within the proton loading network. Small pools of water lead to water exit channels, which in combination with protein sidechains, act as proton exit carriers. Both long-range electrostatic effects (Coulombic interactions) and short-range chemical bonding with charge transfer (for example, Tyr → His → Cu) and proton/electron transfers are critical for all these mechanisms.
3) Cu(I)–histidine bond lability is probably critical to histidine protonation, activating the rapid electron transfer/proton transfer reaction (eT/pT):

$${\text{O}}_{2}^{-}+1e^{-}+1{\text{H}}^{+}\to {\text{OOH}}^{-}\left({\text{Fe}}^{3+}\text{–Superoxide}\to {\text{Fe}}^{3+}\text{–Hydroperoxide, with }1e^{-}{\text{from Cu}}^{+}\right)$$

4) We propose additional mechanisms for transfers of chemical protons into the reaction chamber of the dinuclear center for the proton-coupled electron transfer processes state F → O_H_ and state O_H_ → E_H_. We propose a pumping mechanism in ba_3_ CcO for a single-vector proton via a radical transfer mechanism during the State P_M_ + 1e^−^ → P_R_ transition similar to the one proposed by Yu et al. (2012) in aa_3_-type CcO enzymes.
5) In Complex 3, the splitting (bifurcation) of electron flow between the high potential upper pathway and the low potential lower pathway is controlled by a coupled proton transfer from reduced ubiquinone to the Rieske [2Fe–2S] cluster. In Complex 4, the split pathways (bifurcations) of proton flow depend both on electron transfers, for example, from Cu^2+^ → Cu^+^ (or conversely), on other electron transfers (one electron transfer to superoxide among others), and on the interactions of chemical and vector protons. These comparisons illustrate common themes between Complex 3 and Complex 4 mechanisms, while substantial differences exist in the structures of the metal complexes and their functions, the types of mobile carriers of electrons and protons, and the proton pumping mechanisms.
6) There are many important open questions.
  a. Chemical reaction paths and associated intermediate energies and reaction barriers are incomplete either from an experimental or a quantum chemical/molecular dynamics perspective.
  b. Proton-loading network (PLN) mechanisms and water protein pathways for proton exits are partly developed.
  c. What additional supporting experimental and quantum chemical information can be developed for radical transfer pathways of proton pumping?
  d. Where are reaction mechanisms and energies similar or different in aa_3_-type *versus* ba_3_-type cytochrome c oxidases?
  e. How efficient is the reaction cycle in practice while pumping under an existing membrane potential?
  f. What are the effects of the regulatory subunits in mitochondria? Where are the three core subunits similar or different in aa_3_ CcO enzymes from other organisms (various bacterial aa_3_) or in ba_3_ CcO enzymes?
  g. What are more general themes or insights that we can apply to other biological or chemical systems?

## Data Availability

Publicly available datasets were analyzed in this study. These data can be found in publicly available supplementary material in published scientific journals.

## References

[B1] Badger R. M. (1935). The relation between the internuclear distances and force constants of molecules and its application to polyatomic molecules. J. Chem. Phys. 3, 710–714. 10.1063/1.1749581

[B2] Baerends E. J. Ziegler T. Atkins A. J. Autschbach J. Baseggio O. Bashford D. (2017). SCM, theoretical chemistry. Amsterdam, The Netherlands: Vrije Universiteit. Available at: https://www.scm.com.

[B3] Baymann F. Robertson D. E. Dutton P. L. Mäntele W. (1999). Electrochemical and spectroscopic investigations of the cytochrome bc1 complex from rhodobacter capsulatus. Biochemistry 38, 13188–13199. 10.1021/bi990565b 10529191

[B4] Bloch D. Belevich I. Jasaitis A. Ribacka C. Puustinen A. Verkhovsky M. I. (2004). The catalytic cycle of cytochrome c oxidase is not the sum of its two halves. Proc. Natl. Acad. Sci. 101, 529–533. 10.1073/pnas.0306036101 14699047 PMC327181

[B5] Blomberg M. A. Siegbahn P. E. M. (2012). The mechanism for proton pumping in cytochrome c oxidase from an electrostatic and quantum chemical perspective. Biochimica Biophysica Acta (BBA) - Bioenergetics 1817, 495–505. 10.1016/j.bbabio.2011.09.014 21978537

[B6] Blomberg M. R. A. (2021). The redox-active tyrosine is essential for proton pumping in cytochrome c oxidase. Front. Chem. 9, 640155. 10.3389/fchem.2021.640155 33937193 PMC8079940

[B7] Blomberg M. R. A. Siegbahn P. E. M. (2010). A quantum chemical study of the mechanism for proton-coupled electron transfer leading to proton pumping in cytochrome c oxidase. Mol. Phys. 108, 2733–2743. 10.1080/00268976.2010.523017

[B8] Blomberg M. R. A. Siegbahn P. E. M. Babcock G. T. Wikström M. (2000). Modeling cytochrome oxidase: a quantum chemical study of the o-o bond cleavage mechanism. J. Am. Chem. Soc. 122, 12848–12858. 10.1021/ja002745a

[B9] Cai X. Haider K. Lu J. Radic S. Son C. Y. Cui Q. (2018). Network analysis of a proposed exit pathway for protons to the p-side of cytochrome c oxidase. Biochimica Biophysica Acta (BBA) - 1859, 997–1005. 10.1016/j.bbabio.2018.05.010 29778689

[B10] Cai X. Son C. Y. Mao J. Kaur D. Zhang Y. Khaniya U. (2020). Identifying the proton loading site cluster in the ba_3_ cytochrome c oxidase that loads and traps protons. Biochimica Biophysica Acta (BBA) - Bioenergetics 1861, 148239. 10.1016/j.bbabio.2020.148239 32531221

[B11] Case D. A. Babin V. Berryman J. T. Betz R. M. Cai Q. Cerutti D. S. (2014). AMBER 14. San Francisco, CA: University of California.

[B12] Chang H. Y. Choi S. K. Vakkasoglu A. S. Gennis R. B. Hemp J. Fee J. A. (2012). Exploring the proton pump and exit pathway for pumped protons in cytochrome ba_3_ from thermus thermophilus. Proc. Natl. Acad. Sci. 109, 5259–5264. 10.1073/pnas.1107345109 22431640 PMC3325665

[B13] Chang H. Y. Hemp J. Chen Y. Fee J. A. Gennis R. B. (2009). The cytochrome ba_3_ oxygen reductase from thermus thermophilus uses a single input channel for proton delivery to the active site and for proton pumping. Proc. Natl. Acad. Sci. 106, 16169–16173. 10.1073/pnas.0905264106 19805275 PMC2752507

[B14] Connors K. A. (1990). Chemical kinetics: the study of reaction rates in solution. Wiley.

[B15] de Grotthuss C. (1806). On the decomposition of water and of the bodies that it holds in solution by means of galvanic electricity. J. Ann. Chim. 58, 54–73. 10.1016/j.bbabio.2006.07.004 16962993

[B16] Dickson C. J. Madej B. D. Skjevik Å. A. Betz R. M. Teigen K. Gould I. R. (2014). Lipid14: the Amber lipid force field. J. Chem. Theory Comput. 10, 865–879. 10.1021/ct4010307 24803855 PMC3985482

[B17] Dyer B. D. (2003). A field guide to bacteria. Ithaca, NY: Cornell University Press.

[B18] Egawa T. Chen Y. Fee J. A. Yeh S. R. Rousseau D. L. (2012). The rate-limiting step in O_2_ reduction by cytochrome ba_3_ from thermus thermophilus. Biochimica Biophysica Acta (BBA) - Bioenergetics 1817, 666–671. 10.1016/j.bbabio.2011.11.010 PMC3294028 22138627

[B19] Francia F. Khalfaoui-Hassani B. Lanciano P. Musiani F. Noodleman L. Venturoli G. (2019). The cytochrome b lysine 329 residue is critical for ubihydroquinone oxidation and proton release at the q_0_ site of bacterial cytochrome bc1. Biochimica Biophysica Acta (BBA) - Bioenergetics 1860, 167–179. 10.1016/j.bbabio.2018.12.002 PMC6317905 30550726

[B20] Götz A. W. Williamson M. J. Xu D. Poole D. Le Grand S. Walker R. C. (2012). Routine microsecond molecular dynamics simulations with AMBER on GPUs. 1. Generalized Born. J. Chem. Theory Comput. 8, 1542–1555. 10.1021/ct200909j 22582031 PMC3348677

[B21] Han Du W. G. Götz A. W. Noodleman L. (2018). A water dimer shift activates a proton pumping pathway in the P_r_ → F transition of ba_3_ cytochrome c oxidase. Inorg. Chem. 57, 1048–1059. 10.1021/acs.inorgchem.7b02461 29308889 PMC5825212

[B22] Han Du W. G. Götz A. W. Noodleman L. (2019). DFT Fe_a3_–O/O–O vibrational frequency calculations over catalytic reaction cycle states in the dinuclear center of cytochrome c oxidase. Inorg. Chem. 58, 13933–13944. 10.1021/acs.inorgchem.9b01840 31566371 PMC6839913

[B23] Han Du W. G. Götz A. W. Noodleman L. (2022). DFT calculations for mössbauer properties on dinuclear center models of the resting oxidized cytochrome c oxidase. ChemPhysChem 23, e202100831. 10.1002/cphc.202100831 35142420 PMC9054037

[B24] Han Du W. G. Götz A. W. Yang L. Walker R. C. Noodleman L. (2016). A broken-symmetry density functional study of structures, energies, and protonation states along the catalytic O–O bond cleavage pathway in ba_3_ cytochrome c oxidase from thermus thermophilus. Phys. Chem. Chem. Phys. 18, 21162–21171. 10.1039/C6CP00349D 27094074 PMC4972664

[B25] Han Du W. G. McRee D. Götz A. W. Noodleman L. (2020). A water molecule residing in the Fe_a3_ ^3+^···Cu_B_ ^2+^ dinuclear center of the resting oxidized as-isolated cytochrome *c* oxidase: a density functional study. Inorg. Chem. 59, 8906–8915. 10.1021/acs.inorgchem.0c00724 32525689 PMC8114904

[B26] Hsueh K. L. Westler W. M. Markley J. L. (2010). NMR investigations of the Rieske protein from Thermus thermophilus support a coupled proton and electron transfer mechanism. J. Am. Chem. Soc. 132, 7908–7918. 10.1021/ja1026387 20496909 PMC2882753

[B27] Hunsicker-Wang L. M. Pacoma R. L. Chen Y. Fee J. A. Stout C. D. (2005). A novel cryoprotection scheme for enhancing the diffraction of crystals of recombinant cytochrome ba_3_ oxidase from thermus thermophilus. Acta Crystallogr. D. 61, 340–343. 10.1107/S0907444904033906 15735345

[B28] Ishigami I. Hikita M. Egawa T. Yeh S. R. Rousseau D. L. (2015). Proton translocation in cytochrome c oxidase: insights from proton exchange kinetics and vibrational spectroscopy. Biochimica Biophysica Acta (BBA) - Bioenergetics 1847, 98–108. 10.1016/j.bbabio.2014.09.008 PMC4254173 25268561

[B29] Konecny R. Li J. Fisher C. L. Dillet V. Bashford D. Noodleman L. (1999). CuZn superoxide dismutase geometry optimization, energetics, and redox potential calculations by density functional and electrostatic methods. Inorg. Chem. 38, 940–950. 10.1021/ic980730w 11670866

[B30] Konkle M. E. Elsenheimer K. N. Hakala K. Robicheaux J. C. Weintraub S. T. Hunsicker-Wang L. M. (2010). Chemical modification of the Rieske protein from Thermus thermophilus using diethyl pyrocarbonate modifies ligating histidine 154 and reduces the [2Fe-2S] cluster. Biochemistry 49, 7272–7281. 10.1021/bi1007904 20684561

[B31] Koutsoupakis C. Kolaj-Robin O. Soulimane T. Varotsis C. (2011). Probing protonation/deprotonation of tyrosine residues in cytochrome ba_3_ oxidase from thermus thermophilus by time-resolved step-scan fourier transform infrared spectroscopy. J. Biol. Chem. 286, 30600–30605. 10.1074/jbc.M111.252213 21757723 PMC3162420

[B32] Kuila D. Schoonover J. R. Dyer R. B. Batie C. J. Ballou D. P. Fee J. A. (1992). Resonance Raman studies of Rieske-type proteins. Biochimica Biophysica Acta (BBA) - Bioenergetics 1140, 175–183. 10.1016/0005-2728(92)90007-O 1280165

[B33] Link T. A. Hagen W. R. Pierik A. J. Assmann C. von Jagow G. (1992). Determination of the redox properties of the Rieske [2Fe-2S] cluster of bovine heart bc1 complex by direct electrochemistry of a water-soluble fragment. Eur. J. Biochem. 208, 685–691. 10.1111/j.1432-1033.1992.tb17235.x 1327764

[B34] Maier J. A. Martinez C. Kasavajhala K. Wickstrom L. Hauser K. E. Simmerling C. (2015). ff14SB: Improving the Accuracy of Protein Side Chain and Backbone Parameters from ff99SB. J. Chem. Theory Comput. 11, 3696–3713. 10.1021/acs.jctc.5b00255 26574453 PMC4821407

[B35] Makhov D. V. Popović D. M. Stuchebrukhov A. A. (2006). Improved density functional theory/electrostatic calculation of the His291 protonation state in cytochrome C oxidase: self-consistent charges for solvation energy calculation. J. Phys. Chem. B 110, 12162–12166. 10.1021/jp0608630 16800531

[B36] Mayer I. (1983). Charge, bond order and valence in the *ab initio* SCF theory. Chem. Phys. Lett. 97, 270–274. 10.1016/0009-2614(83)80005-0

[B37] Nicholls D. G. Ferguson S. J. (2002). Bioenergetics. San Diego: Academic Press.

[B38] Noodleman L. Han Du W. G. Fee J. A. Götz A. W. Walker R. C. (2014). Linking chemical electron–proton transfer to proton pumping in cytochrome c oxidase: broken-symmetry DFT exploration of intermediates along the catalytic reaction pathway of the iron–copper dinuclear complex. Inorg. Chem. 53, 6458–6472. 10.1021/ic500363h 24960612 PMC4095914

[B39] Noodleman L. Han Du W. G. McRee D. Chen Y. Goh T. Götz A. W. (2020). Coupled transport of electrons and protons in a bacterial cytochrome c oxidase – DFT calculated properties compared to structures and spectroscopies. Phys. Chem. Chem. Phys. 22, 26652–26668. 10.1039/D0CP04848H 33231596 PMC7727307

[B40] Perry J. Shin D. Getzoff E. Tainer J. (2010). The structural biochemistry of the superoxide dismutases. Biochimica Biophysica Acta (BBA) - Proteins Proteomics 1804, 245–262. 10.1016/j.bbapap.2009.11.004 PMC3098211 19914407

[B41] Poiana F. von Ballmoos C. Gonska N. Blomber M. R. A. Ädelroth P. Brzezinski P. (2017). Splitting of the o–o bond at the heme-copper catalytic site of respiratory oxidases. Sci. Adv. 3, e1700279. 10.1126/sciadv.1700279 28630929 PMC5473675

[B42] Popović D. M. (2013). Current advances in research of cytochrome c oxidase. Amino Acids 45, 1073–1087. 10.1007/s00726-013-1585-y 23999646

[B43] Popović D. M. Quenneville J. Stuchebrukhov A. A. (2005). Dft/electrostatic calculations of pk_a_ values in cytochrome c oxidase. J. Phys. Chem. B 109, 3616–3626. 10.1021/jp046535m 16851400

[B44] Popović D. M. Stuchebrukhov A. A. (2004). Electrostatic study of the proton pumping mechanism in bovine heart cytochrome c oxidase. J. Am. Chem. Soc. 126, 1858–1871. 10.1021/ja038267w 14871119

[B45] Popović D. M. Stuchebrukhov A. A. (2005). Proton exit channels in bovine cytochrome c oxidase. J. Phys. Chem. B 109, 1999–2006. 10.1021/jp0464371 16851184

[B46] Radoń M. Pierloot K. (2008). Binding of co, no, and o2 to heme by density functional and multireference *ab initio* calculations. J. Phys. Chem. A 112, 11824–11832. 10.1021/jp806075b 18942804

[B47] Rauhamäki V. Wikström M. (2014). The causes of reduced proton-pumping efficiency in type b and c respiratory heme-copper oxidases, and in some mutated variants of type a. Biochimica Biophysica Acta (BBA) - Bioenergetics 1837, 999–1003. 10.1016/j.bbabio.2014.02.020 24583065

[B48] Rouault T. A. Tong W. H. (2008). Iron–sulfur cluster biogenesis and human disease. Trends Genet. 24, 398–407. 10.1016/j.tig.2008.05.008 18606475 PMC2574672

[B49] Salomon-Ferrer R. Case D. A. Walker R. C. (2013a). An overview of the Amber biomolecular simulation package. WIREs Comput. Mol. Sci. 3, 198–210. 10.1002/wcms.1121

[B50] Salomon-Ferrer R. Götz A. W. Poole D. Le Grand S. Walker R. C. (2013b). Routine microsecond molecular dynamics simulations with AMBER on GPUs. 2. Explicit solvent particle mesh Ewald. J. Chem. Theory Comput. 9, 3878–3888. 10.1021/ct400314y 26592383

[B51] Shin D. S. DiDonato M. Barondeau D. P. Hura G. L. Hitomi C. Berglund J. A. (2009). Superoxide dismutase from the eukaryotic thermophile alvinella pompejana: structures, stability, mechanism, and insights into amyotrophic lateral sclerosis. J. Mol. Biol. 385, 1534–1555. 10.1016/j.jmb.2008.11.031 19063897 PMC2669833

[B52] Siegbahn P. E. M. Blomberg M. R. A. Blomberg M. L. (2003). Theoretical study of the energetics of proton pumping and oxygen reduction in cytochrome oxidase. J. Phys. Chem. B 107, 10946–10955. 10.1021/jp035486v

[B53] Siletsky S. A. Belevich I. Jasaitis A. Konstantinov A. A. Wikström M. Soulimane T. (2007). Time-resolved single-turnover of ba3 oxidase from thermus thermophilus. Biochimica Biophysica Acta (BBA) - Bioenergetics 1767, 1383–1392. 10.1016/j.bbabio.2007.09.010 17964277

[B54] Spassov V. Z. Luecke H. Gerwert K. Bashford D. (2001). Pk_a_ calculations suggest storage of an excess proton in a hydrogen-bonded water network in bacteriorhodopsin. J. Mol. Biol. 312, 203–219. 10.1006/jmbi.2001.4902 11545597

[B55] Sugitani R. Stuchebrukhov A. A. (2009). Molecular dynamics simulation of water in cytochrome c oxidase reveals two water exit pathways and the mechanism of transport. Biochimica Biophysica Acta (BBA) - Bioenergetics 1787, 1140–1150. 10.1016/j.bbabio.2009.04.004 PMC4220738 19393218

[B56] Szundi I. Funatogawa C. Fee J. A. Soulimane T. Einarsdóttir O. (2010). CO impedes superfast O_2_ binding in ba_3_ cytochrome oxidase from thermus thermophilus. Proc. Natl. Acad. Sci. 107, 21010–21015. 10.1073/pnas.1008603107 21097703 PMC3000243

[B57] te Velde G. Bickelhaupt F. M. Baerends E. J. Fonseca Guerra C. van Gisbergen S. J. A. Snijders J. G. (2001). Chemistry with ADF. J. Comput. Chem. 22, 931–967. 10.1002/jcc.1056

[B58] Tiefenbrunn T. Liu W. Chen Y. Katritch V. Stout C. D. Fee J. A. (2011). High resolution structure of the ba_3_ cytochrome c oxidase from thermus thermophilus in a lipidic environment. PLOS ONE 6, 223488–e22412. 10.1371/journal.pone.0022348 PMC3141039 21814577

[B59] Ullmann M. G. Noodleman L. Case D. A. (2002). Density functional calculation of pKa values and redox potentials in the bovine Rieske iron-sulfur protein. J. Biol. Inorg. Chem. 7, 632–639. 10.1007/s00775-002-0342-6 12072969

[B60] Vancoillie S. Zhao H. Radoń M. Pierloot K. (2010). Performance of caspt2 and dft for relative spin-state energetics of heme models. J. Chem. Theory Comput. 6, 576–582. 10.1021/ct900567c 26617311

[B61] van Lenthe E. Baerends E. J. (2003). Optimized Slater-type basis sets for the elements 1-118. J. Comput. Chem. 24, 1142–1156. 10.1002/jcc.10255 12759913

[B62] Wikström M. Gennis R. B. Rich P. R. (2023). Structures of the intermediates in the catalytic cycle of mitochondrial cytochrome c oxidase. Biochimica Biophysica Acta (BBA) - Bioenergetics 1864, 148933. 10.1016/j.bbabio.2022.148933 36403794

[B63] Wikström M. Krab K. Sharma V. (2018). Oxygen activation and energy conservation by cytochrome c oxidase. Chem. Rev. 118, 2469–2490. 10.1021/acs.chemrev.7b00664 29350917 PMC6203177

[B64] Yang L. Skjevik Å. A. Han Du W. G. Noodleman L. Walker R. C. Götz A. W. (2016a). Water exit pathways and proton pumping mechanism in B-type cytochrome c oxidase from molecular dynamics simulations. Biochimica Biophysica Acta (BBA) - Bioenergetics 1857, 1594–1606. 10.1016/j.bbabio.2016.06.005 PMC4995112 27317965

[B65] Yang L. Skjevik A. A. Han Du W. G. Noodleman L. Walker R. C. Götz A. W. (2016b). Data for molecular dynamics simulations of B-type cytochrome c oxidase with the amber force field. Data Brief 8, 1209–1214. 10.1016/j.dib.2016.07.043 27547799 PMC4979044

[B66] Yang X. Liu S. Yin Z. Chen M. Song J. Li P. (2023). New insight into the proton pumping mechanism of ba_3_ cytochrome c oxidase: the functions of key residues and waters. Phys. Chem. Chem. Phys. 25, 25105–25115. 10.1039/D3CP01334K 37461851

[B67] Yu M. A. Egawa T. Shinzawa-Itoh K. Yoshikawa S. Guallar V. Yeh S. R. (2012). Two tyrosyl radicals stabilize high oxidation states in cytochrome c oxidase for efficient energy conservation and proton translocation. J. Am. Chem. Soc. 134, 4753–4761. 10.1021/ja210535w 22296274 PMC3418888

[B68] Zu Y. Couture M. M. J. Kolling D. R. J. Crofts A. R. Eltis L. D. Fee J. A. (2003). Reduction potentials of Rieske clusters: importance of the coupling between oxidation state and histidine protonation state. Biochemistry 42, 12400–12408. 10.1021/bi0350957 14567701

[B69] Zu Y. Fee J. A. Hirst J. (2001). Complete thermodynamic characterization of reduction and protonation of the bc1-type Rieske [2Fe-2S] center of Thermus thermophilus. J. Am. Chem. Soc. 123, 9906–9907. 10.1021/ja016532c 11583559

